# Comparison of in silico predictions of action potential duration in response to inhibition of I_Kr_ and I_CaL_ with new human ex vivo recordings

**DOI:** 10.1371/journal.pcbi.1012913

**Published:** 2025-07-07

**Authors:** Yann-Stanislas H. M. Barral, Liudmila Polonchuk, Michael Clerx, David J. Gavaghan, Gary R. Mirams, Ken Wang

**Affiliations:** 1 Roche Pharma Research and Early Development, Pharmaceutical Sciences, Roche Innovation Center Basel, F. Hoffmann-La Roche Ltd., Basel, Switzerland; 2 Department of Computer Science, University of Oxford, Oxford, United Kingdom; 3 Centre for Mathematical Medicine and Biology, School of Mathematical Sciences, University of Nottingham, Nottingham, United Kingdom; University of Bath, UNITED KINGDOM OF GREAT BRITAIN AND NORTHERN IRELAND

## Abstract

During drug development, candidate compounds are extensively tested for proarrhythmic risk and in particular risk of Torsade de Pointes (TdP), as indicated by prolongation of the QT interval. Drugs that inhibit the rapid delayed rectifier ${\text{K}}^{+}$ current ($I_{\mathrm{Kr}}$) can prolong the action potential duration (APD) and thereby the QT interval, and so are routinely rejected. However, simultaneous inhibition of the L-type ${\text{Ca}}^{2+}$ current ($I_{\mathrm{CaL}}$) can mitigate the effect of $I_{\mathrm{Kr}}$ inhibition, so that including both effects can improve test specificity. Mathematical models of the action potential (AP) can be used to predict the APD prolongation resulting from a given level of $I_{\mathrm{Kr}}$ and $I_{\mathrm{CaL}}$ inhibition, but for use in safety-testing their predictive capabilities should first be carefully verified. We present the first systematic comparison between experimental drug-induced APD and predictions by AP models. New experimental data were obtained *ex vivo* for APD response to $I_{\mathrm{Kr}}$ and/or $I_{\mathrm{CaL}}$ inhibition by applying 9 compounds at different concentrations to adult human ventricular trabeculae at physiological temperature. Compounds with similar effects on $I_{\mathrm{Kr}}$ and $I_{\mathrm{CaL}}$ exhibited less APD prolongation compared to selective $I_{\mathrm{Kr}}$ inhibitors. We then integrated *in vitro* ${\text{IC}}_{50}$ patch-clamp data for $I_{\mathrm{Kr}}$ and $I_{\mathrm{CaL}}$ inhibition by the tested compounds into simulations with AP models. Models were assessed against the *ex vivo* data on their ability to recapitulate drug-induced APD changes observed experimentally. None of the tested AP models reproduced the APD changes observed experimentally across all combinations and degrees of $I_{\mathrm{Kr}}$ and/or $I_{\mathrm{CaL}}$ inhibition: they matched the data either for selective $I_{\mathrm{Kr}}$ inhibitors or for compounds with comparable effects on $I_{\mathrm{Kr}}$ and $I_{\mathrm{CaL}}$. This work introduces a new benchmarking framework to assess the predictivity of current and future AP models for APD response to $I_{\mathrm{Kr}}$ and/or $I_{\mathrm{CaL}}$ inhibition. This is an essential primary step towards an *in silico* framework that integrates *in vitro* data for translational clinical cardiac safety.

## Author summary

Before an investigational drug reaches patients, it is tested *in vitro* to ensure it does not disrupt the heart’s electric activity. This testing often focuses on the drug’s ability to block a specific current called $I_{\mathrm{Kr}}$, which, if inhibited, can prolong the heart cells’ action potential duration (APD), which is associated with an increased risk of irregular heartbeats (proarrhythmia). Our study examines how blocking another current, $I_{\mathrm{CaL}}$, along with $I_{\mathrm{Kr}}$, affects APD. We found that adding $I_{\mathrm{CaL}}$ inhibition may mitigate the proarrhythmic effects caused by $I_{\mathrm{Kr}}$ inhibition alone. Understanding this balance can improve how we assess the cardiac safety of new drugs, potentially saving promising compounds from being incorrectly discarded. Currently, mathematical models help predict such cardiac responses, but no existing model accurately predicted our findings. Our new data could aid in developing more predictive models in the future. This will contribute to safer drug development and more effective treatments.

## 1. Introduction

The rapid delayed rectifier ${\text{K}}^{+}$ current ($I_{\mathrm{Kr}}$) is a major ionic current responsible for the repolarisation of ventricular cardiomyocytes [1]. Inhibition of $I_{\mathrm{Kr}}$ prolongs the action potential (AP) duration (APD) and the QT interval [2]. Many drugs inhibiting $I_{\mathrm{Kr}}$ have been shown to increase the risk of Torsade de Pointes (TdP), a potentially deadly arrhythmia [2,3]. Regulatory bodies established guidelines ICH S7B and ICH E14 to prevent the development of new compounds with unacceptable pro-arrhythmic risk [4,5]. According to ICH S7B, the ability of compounds to inhibit $I_{\mathrm{Kr}}$ should be tested *in vitro*. Redfern *et al*. suggested a “safety margin” such that drugs should have a half-maximal inhibitory concentration (${\text{IC}}_{50}$) of over 30 times their maximal free therapeutic plasma concentration [2].

Multiple ion channels affect the TdP risk, notably the inhibition of the L-type ${\text{Ca}}^{2+}$ current ($I_{\mathrm{CaL}}$) mitigates the arrhythmogenicity of $I_{\mathrm{Kr}}$ inhibitors [6]. AP models can improve the limited specificity of $I_{\mathrm{Kr}}$–centric TdP risk assessment by accounting for simultaneous inhibition of multiple ionic currents [7]. The Comprehensive in Vitro Proarrhythmia Assay (CiPA) initiative has encouraged the adoption of biophysically-detailed mathematical AP models as a framework to integrate *in vitro* ion channel data and assess drug-induced TdP risk [8]. Yet, AP model predictions of APD changes induced by simultaneous inhibition of $I_{\mathrm{Kr}}$ and $I_{\mathrm{CaL}}$ have not been validated against human data.

In this study, we measure *ex vivo* the APD at 90% repolarisation (${\text{APD}}_{90}$) in human adult ventricular trabeculae, with inhibition of $I_{\mathrm{Kr}}$ and/or $I_{\mathrm{CaL}}$ by 9 compounds (Chlorpromazine, Clozapine, Dofetilide, Fluoxetine, Mesoridazine, Nifedipine, Quinidine, Thioridazine, Verapamil). For each compound, we subsequently use patch clamp data to calculate the percentage of block of $I_{\mathrm{Kr}}$ and/or $I_{\mathrm{CaL}}$ at the compound concentrations in trabeculae experiments. These numbers are then used as inputs into AP simulations to compare the predictions of 11 *in silico* AP models with the *ex vivo* data.

${\text{APD}}_{90}$ changes from baseline ($\Delta {\text{APD}}_{90}$) induced by $I_{\mathrm{Kr}}$ and $I_{\mathrm{CaL}}$ inhibition are linked to QT changes [9]. By comparing predictions by existing AP models with the *ex vivo* data, we assess their predictivity in a context relevant to drug development. We thus introduce a benchmarking framework to validate current and future AP models. Therefore, this work can be re-used to help develop predictive models for QT change induced by $I_{\mathrm{Kr}}$ and/or $I_{\mathrm{CaL}}$ inhibition.

## 2. Results

### 2.1. Experimental change in ${\text{APD}}_{90}$ from baseline with drug exposure

Experimental ${\text{APD}}_{90}$ measured after 25 min of steady 1 Hz pacing are summarised in Table 1 for the 9 tested compounds. The standard error of the mean (SEM) is also reported in the Table 1.

**Table 1:** A summary of trabeculae recordings for average ${\text{APD}}_{90}$ at baseline and drug-induced ${\text{APD}}_{90}$ change from baseline ($\Delta {\text{APD}}_{90}$). SEM: Standard error of the mean. SD: Standard deviation.

Drug	Mean baseline ${\text{APD}}_{90}$ (SD) in ms	Nominal drug conc (μM)	Mean $\Delta {\text{APD}}_{90}$ (SEM), in ms
Chlorpromazine	299 (36)	0.3	+ 9 (10)
		1	+ 18 (8)
		3	+ 24 (11)
Clozapine	324 (51)	0.3	+ 8 (5)
		1	+ 10 (7)
		3	+ 10 (7)
		30	+ 15 (9)
Dofetilide	317 (51)	0.001	+ 20 (5)
		0.01	+ 82 (8)
		0.1	+ 256 (21)
		0.2	+ 318 (33)
Fluoxetine	271 (36)	0.3	+ 10 (4)
		1	+ 6 (7)
		3	–2 (5)
Mesoridazine	334 (60)	0.04	–2 (7)
		0.25	+ 2 (4)
		10	+ 21 (2)
Nifedipine	336 (55)	0.003	+ 7 (4)
		0.03	–5 (6)
		0.3	–24 (6)
Quinidine	302 (53)	0.1	+ 6 (5)
		1	+ 8 (5)
		10	+ 37 (7)
Thioridazine	307 (48)	0.012	+ 15 (9)
		0.6	+ 16 (20)
		2	+ 6 (14)
Verapamil	349 (61)	0.01	–15 (4)
		0.1	–19 (5)
		1	–20 (10)

Chlorpromazine, Clozapine, Fluoxetine, and Mesoridazine induced little or no change in ${\text{APD}}_{90}$  as the effects of $I_{\mathrm{Kr}}$ and $I_{\mathrm{CaL}}$ inhibition on ${\text{APD}}_{90}$ compensated each other. Verapamil, whilst exhibiting similar effects on $I_{\mathrm{Kr}}$ and $I_{\mathrm{CaL}}$, substantially shortened ${\text{APD}}_{90}$ (–15 ms to –20 ms on average) with high variability in $\Delta {\text{APD}}_{90}$ (SEM up to 35 ms).

Substantial variability of baseline ${\text{APD}}_{90}$ was observed in trabeculae tested with Mesoridazine, Clozapine, and Nifedipine (SD of 60 ms, 51 ms, and 55 ms respectively), but this did not lead to particularly high variability in drug induced $\Delta {\text{APD}}_{90}$. The SEM of $\Delta {\text{APD}}_{90}$ was below 7 ms, 9 ms, and 6 ms for Mesoridazine, Clozapine, and Nifedipine, respectively. Fluoxetine-induced $\Delta {\text{APD}}_{90}$ also exhibited low SEM (≤7 ms). In contrast, Dofetilide induced the most variable $\Delta {\text{APD}}_{90}$  with up to 33 ms SEM for 200 nM Dofetilide.

**Fig 1:**
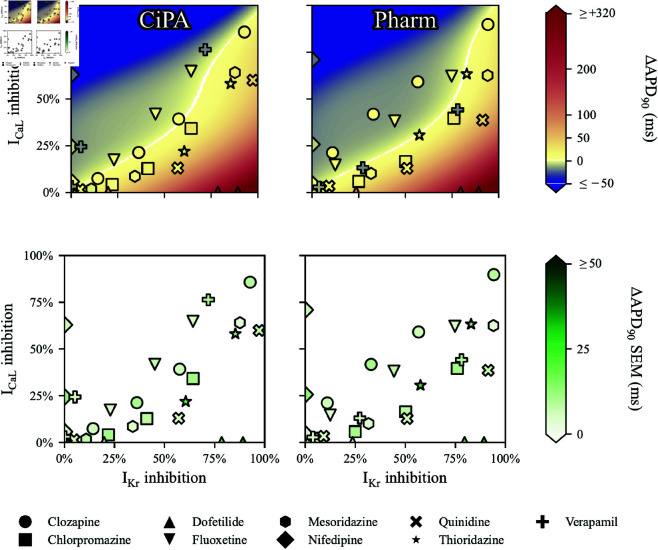
Experimental $\Delta {\text{APD}}_{90}$ measured ex vivo under various drug conditions in human ventricular trabeculae, as a function of $I_{\mathrm{Kr}}$ and $I_{\mathrm{CaL}}$ inhibition and cubic surface approximating the trabeculae data points in the background. $I_{\mathrm{Kr}}$ and $I_{\mathrm{CaL}}$ inhibition were computed using the Hill equation (Eq 1), with the CiPA (left) and Pharm (right) datasets. The bottom panels report the inter-trabeculae variability observed experimentally. The marker type indicates the drug used to apply the $I_{\mathrm{Kr}}$ and $I_{\mathrm{CaL}}$ inhibition.

The 2-D maps of experimental $\Delta {\text{APD}}_{90}$ are plotted in Fig 1, with $I_{\mathrm{Kr}}$ and $I_{\mathrm{CaL}}$ inhibition computed with both the CiPA and Pharm datasets.

With increasing $I_{\mathrm{CaL}}$ inhibition, ${\text{APD}}_{90}$ was shortened (shown as darker blue colors). On the other hand, the more $I_{\mathrm{Kr}}$ was inhibited, the more ${\text{APD}}_{90}$ was prolonged. $I_{\mathrm{Kr}}$ and $I_{\mathrm{CaL}}$ inhibition differed from one dataset to another, with the CiPA dataset exhibiting more sensitivity to inhibition of $I_{\mathrm{CaL}}$ than the Pharm dataset.

Note the biggest outlier from the surface, where 1 μM Verapamil induced $\Delta {\text{APD}}_{\text{90}}=-20\pm 10$ ms at 1 μM, with 78% $I_{\mathrm{Kr}}$ and 44% $I_{\mathrm{CaL}}$ inhibition. For comparison, 3 μM Clozapine induced $\Delta {\text{APD}}_{\text{90}}=+10\pm 7$ ms with 57% $I_{\mathrm{Kr}}$ and 59% $I_{\mathrm{CaL}}$ inhibition.

### 2.2. 2-D maps of $\Delta {\text{APD}}_{90}$ predicted by literature AP models

**Fig 2:**
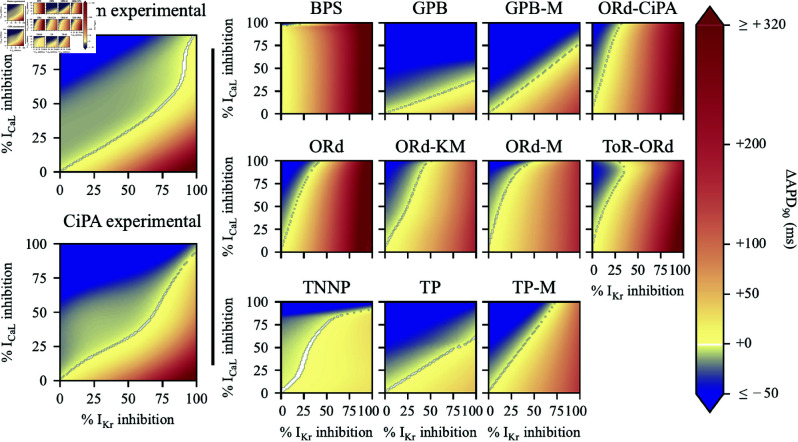
Left: Surfaces fitted through experimental data points. **Right:** 2-D maps of predicted ${\text{APD}}_{90}$ change from baseline after $I_{\mathrm{CaL}}$ and $I_{\mathrm{Kr}}$ inhibition. The colour scale indicates shortening of ${\text{APD}}_{90}$ (i.e., $\Delta {\text{APD}}_{90}$ < 0 ms) for colours towards dark blue, and ${\text{APD}}_{90}$ prolongation (i.e., $\Delta {\text{APD}}_{90}$ > 0 ms) for colours towards red. $\Delta {\text{APD}}_{90}$ values below –50 ms and above  + 320 ms were set to dark blue and red, respectively, for better visualisation. For $I_{\mathrm{Kr}}$ and $I_{\mathrm{CaL}}$ inhibition leading to $-1\;\text{ms}<\Delta {\text{APD}}_{90}<+1$ ms, the pixel is coloured in white.

The 2-D maps of $\Delta {\text{APD}}_{90}$ prediction for all 11 models and variants are shown in Fig 2 with cubic surfaces fitted through experimental data points.

A clear distinction was observed between models similar to the ORd model, which were most sensitive to $I_{\mathrm{Kr}}$ inhibition, and TP-like models, which were more sensitive to $I_{\mathrm{CaL}}$ inhibition. On 2-D maps for the BPS, ORd, ORd-CiPA, ORd-KM, ORd-M, and ToR-ORd models, the 0 ms line was mostly vertical, indicating little mitigation of $I_{\mathrm{Kr}}$ inhibition-induced $\Delta {\text{APD}}_{90}$ by $I_{\mathrm{CaL}}$ inhibition. These results align with previous observations [10].

In the BPS model, nearly no mitigation of $I_{\mathrm{Kr}}$ inhibition by $I_{\mathrm{CaL}}$ inhibition was observed, and the 0 ms line was vertical. $I_{\mathrm{CaL}}$ inhibition even prolonged ${\text{APD}}_{90}$: 5% $I_{\mathrm{Kr}}$ and 80% $I_{\mathrm{CaL}}$ inhibition yielded $\Delta {\text{APD}}_{\text{90}}=+9$ ms, whilst 5% $I_{\mathrm{Kr}}$ and 85% $I_{\mathrm{CaL}}$ inhibition yielded $\Delta {\text{APD}}_{\text{90}}=+11$ ms. $\Delta {\text{APD}}_{90}$ predicted by the BPS model was not monotonic. Initially, $I_{\mathrm{CaL}}$ inhibition prolonged ${\text{APD}}_{90}$, but with more than 91% $I_{\mathrm{CaL}}$ inhibition, ${\text{APD}}_{90}$ decreased drastically.

The ToR-ORd model also exhibited a non-monotonic 2-D map: for 35% $I_{\mathrm{Kr}}$ and 90% $I_{\mathrm{CaL}}$ inhibition, no change in APD90 was predicted; further $I_{\mathrm{CaL}}$ inhibition increased ${\text{APD}}_{90}$. In simulations, the strongly reduced $I_{\mathrm{CaL}}$ shrinks the ${\text{Ca}}^{2+}$ concentration in the subspace compartment, reducing the repolarising calcium-activated ${\text{Cl}}^{-}$- current (${\text{I}}_{\text{(Ca)Cl}}$), and therefore prolonging ${\text{APD}}_{90}$.

The TP-like models (TP, TP-M, GPB, and GPB-M) predicted similar 0 ms lines, almost linear with slopes between 0.5 and 1.3. The original TP and GPB models exhibited much lower sensitivities of ${\text{APD}}_{90}$ to selective $I_{\mathrm{Kr}}$ inhibition ($\Delta {\text{APD}}_{\text{90}}\leq +48$ ms and  + 51 ms respectively), than observed experimentally with 200 nM Dofetilide ($\Delta {\text{APD}}_{\text{90}}=+318\pm 33$ ms). Adjustments by Mann *et al*. increased their sensitivity to $I_{\mathrm{Kr}}$ inhibition [11] : the TP-M and GPB-M models predicted $\Delta {\text{APD}}_{\text{90}}=+154$ ms and  + 144 ms with 100% $I_{\mathrm{Kr}}$ inhibition, respectively.

The TNNP model behaved differently, with its 0 ms line in an “S” shape, and nearly no prolongation of ${\text{APD}}_{90}$, even with 100% $I_{\mathrm{Kr}}$ inhibition ($\Delta {\text{APD}}_{\text{90}}<+30$ ms).

Visually comparing model predictions with *ex vivo* data, the TP-M and GPB-M models appear closest to the truth. This is quantitatively investigated in the next section.

### 2.3. Comparison of in-silico prediction of $\Delta {\text{APD}}_{90}$ with ex vivo data

**Fig 3:**
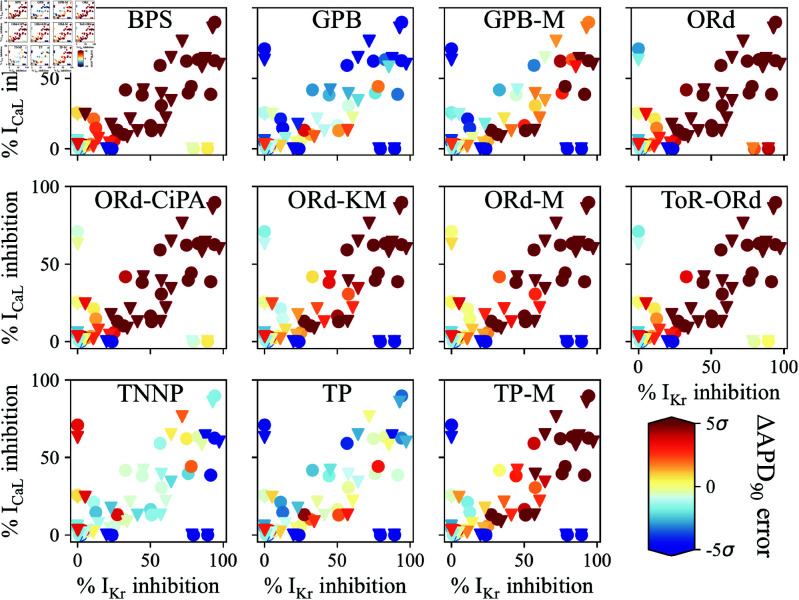
Comparison of in-silico prediction of $\Delta {\text{APD}}_{90}$ response to $I_{\mathrm{Kr}}$ and/or $I_{\mathrm{CaL}}$ inhibition with ex vivo data. CiPA (**triangle**) and Pharm (**circle**) datasets for ${\text{IC}}_{50}$ values were used to compute drug perturbation. $\sigma_{\text{M}}$ denotes here the experimental standard error of the mean $\Delta {\text{APD}}_{90}$ response to each drug perturbation.

Fig 3 presents the quantitative comparison of the *ex vivo* data with $\Delta {\text{APD}}_{90}$ predictions, using external ionic concentrations in simulations that matched the experimental settings. The error in $\Delta {\text{APD}}_{90}$ is shown as a multiple of the experimental SEM in $\Delta {\text{APD}}_{90}$ ($\sigma_{\text{M}}$), directly visualising each condition’s contribution to the error measure, *E* (Eq 2).

The ORd-like models performed similarly in predicting experimental $\Delta {\text{APD}}_{90}$  consistently with their 2-D maps (Fig 2). Predictions for selective $I_{\mathrm{Kr}}$ and $I_{\mathrm{CaL}}$ inhibitors by the ORd-CiPA and ToR-ORd models were largely correct (light colors). However, ORd-like models overpredicted ${\text{APD}}_{90}$ prolongation induced by simultaneous $I_{\mathrm{Kr}}$ and $I_{\mathrm{CaL}}$ inhibition (dark red).

The TP-like models underpredicted the $\Delta {\text{APD}}_{90}$ response to selective $I_{\mathrm{Kr}}$ inhibition (blue) but provided good predictions for mitigation by $I_{\mathrm{CaL}}$ inhibition. The GPB model predicted excessive ${\text{APD}}_{90}$ shortening after more than 50% $I_{\mathrm{CaL}}$ inhibition, but its predictions for simultaneous inhibition of $I_{\mathrm{Kr}}$ and $I_{\mathrm{CaL}}$ were within $3\;\times \;\sigma_{\text{M}}$. The GPB-M and TP-M models overpredicted ${\text{APD}}_{90}$ prolongation induced by simultaneous $I_{\mathrm{Kr}}$ and $I_{\mathrm{CaL}}$ inhibition, depending on the ${\text{IC}}_{50}$ dataset. An alternative visualisation of performances of the various models is provided in S1 Appendix.

**Fig 4:**
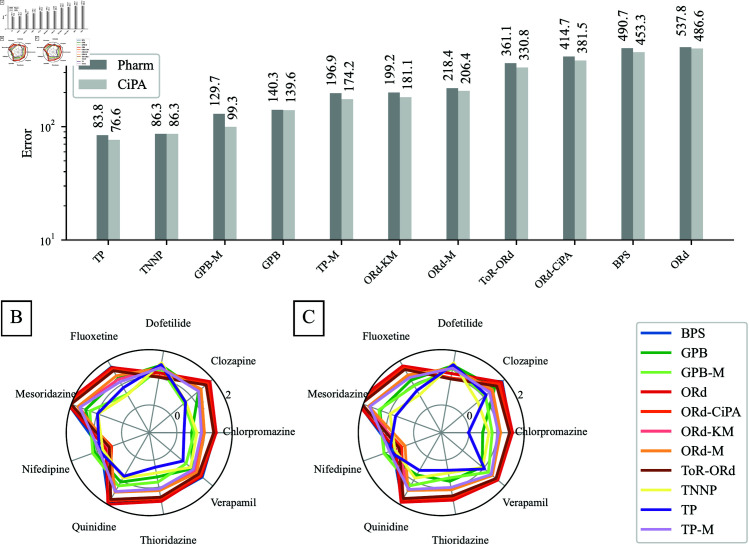
Comparison of the abilities of human ventricular AP models to reproduce the ${\text{APD}}_{90}$ response to $I_{\mathrm{Kr}}$ and $I_{\mathrm{CaL}}$ inhibition observed ex vivo. The lower the error measure (Eq 2), the more accurate the model predictions. **A:** The error measure was summed over all the drugs used in this study, when using the CiPA and Pharm protocols to compute the reduction of ionic currents by drugs. For each model, two bar plots were plotted, to compare the predictive power of models with the Pharm (left bar) and the CiPA (right bar) datasets. **B and C:** Detail of the error measures associated with each of the drugs using the CiPA and Pharm datasets, respectively, for each model. The log_10_ of the error measure is plotted along the radial-axis.

Fig 4A compares *E* (Eq 2) for all 10 models using the CiPA and Pharm datasets. Fig 4B and 4C detail *E* for each drug.

The TP model yielded the lowest errors *E* = 76.6 and 83.8 using the CiPA and Pharm datasets, respectively. Low errors were found for all drugs with similar effects on $I_{\mathrm{Kr}}$ and $I_{\mathrm{CaL}}$. The largest *E* for the TP model were for Dofetilide (24.2–31.2) and Nifedipine (8.3–9.8). All TP-like models showed high *E* for Dofetilide and Nifedipine, consistent with Fig 3, where the largest *E* was for selective $I_{\mathrm{Kr}}$ or $I_{\mathrm{CaL}}$ inhibition.

For the TP and GPB models, *E* computed using the two datasets did not differ significantly. In contrast, models reformulated by Mann *et al*. [11] and ORd-like models showed stronger dependency on the dataset. A difference of 51.2 was obtained between the CiPA and Pharm datasets with the ORd model: *E* = 486.6 versus *E* = 537.8, respectively.

ORd-like models performed similarly, with low errors for Dofetilide and Nifedipine but high errors for other drugs (up to 193.1 for Mesoridazine with the Pharm dataset for the ORd model). These models reproduced the ${\text{APD}}_{90}$ response to selective $I_{\mathrm{Kr}}$ or $I_{\mathrm{CaL}}$ inhibition well but did not capture the mitigation of $I_{\mathrm{Kr}}$ inhibition by $I_{\mathrm{CaL}}$ inhibition.

## 3. Discussion

### 3.1. Main findings

The performance of 11 literature AP models was evaluated against new *ex vivo* data from adult human ventricular trabeculae, which measured ${\text{APD}}_{90}$ response to inhibition of $I_{\mathrm{Kr}}$ and/or $I_{\mathrm{CaL}}$ by 9 different drugs.

The TP-like models exhibited less sensitivity to $I_{\mathrm{Kr}}$ inhibition compared to the ORd-like models. The error measure for $\Delta {\text{APD}}_{90}$ prediction, *E* (Eq 2), was lower with the TP-like models, with the lowest error obtained for the TP model using the Pharm dataset. Their predictions are closer to experimental values for the mitigation of ${\text{APD}}_{90}$ response to $I_{\mathrm{Kr}}$ inhibition by $I_{\mathrm{CaL}}$ inhibition, but are less accurate for selective $I_{\mathrm{Kr}}$ and $I_{\mathrm{CaL}}$ inhibitors.

The opposite was observed with ORd-like models. They make accurate predictions of ${\text{APD}}_{90}$ response to selective $I_{\mathrm{Kr}}$ inhibition, but do not capture the mitigating effect of $I_{\mathrm{CaL}}$ inhibition on $I_{\mathrm{Kr}}$ inhibition-induced ${\text{APD}}_{90}$ prolongation.

Mann *et al*. demonstrated that rescaling maximal conductance parameters of the TP and GPB models and adding a component for ${\text{I}}_{\text{NaL}}$ can increase their sensitivity to $I_{\mathrm{Kr}}$ inhibition whilst preserving the compensating effects of $I_{\mathrm{CaL}}$ and $I_{\mathrm{Kr}}$ inhibition on $\Delta {\text{APD}}_{90}$ [11].

In summary, our novel data can be used as a benchmark to assess the predictivity of any future AP model for $\Delta {\text{APD}}_{90}$ response to $I_{\mathrm{Kr}}$ and/or $I_{\mathrm{CaL}}$ inhibition. Of the currently available models we selected, the TP and GPB-M models showed better overall performance. They therefore appear as promising base models for predicting $\Delta {\text{APD}}_{90}$ response to multi-ion channel inhibitors and subsequent QT changes, upon further development.

### 3.2. Model differences

Several models in this study were validated against previous experimental data for ${\text{APD}}_{90}$ prolongation following $I_{\mathrm{Kr}}$ inhibition [11–16]. ${\text{APD}}_{90}$ shortening with selective $I_{\mathrm{CaL}}$ inhibition was also included in the development of the BPS, ORd, and ToR-ORd models. For instance, the ORd model’s $\Delta {\text{APD}}_{90}$ predictions were validated for ${\text{APD}}_{90}$ response to 70% $I_{\mathrm{Kr}}$ inhibition in guinea pig cardiomyocytes [17] and to 90% $I_{\mathrm{CaL}}$ inhibition in rat cardiomyocytes [18]. whilst the model qualitatively agrees with experimental ${\text{APD}}_{90}$ responses to selective $I_{\mathrm{Kr}}$ and $I_{\mathrm{CaL}}$ inhibitors, ORd-like models fail to predict $\Delta {\text{APD}}_{90}$ for simultaneous $I_{\mathrm{Kr}}$ and $I_{\mathrm{CaL}}$ inhibition. Predictions show an $I_{\mathrm{Kr}}$-dominated prolongation where *ex vivo* data show mitigation by $I_{\mathrm{CaL}}$ inhibition.

This emphasises the need for context-specific model validation [19]. For example, the ORd-CiPA model, validated for TdP risk classification [20], tends to overestimate APD response to simultaneous $I_{\mathrm{Kr}}$ and $I_{\mathrm{CaL}}$ inhibition. Similarly, the BPS model, validated for 100% $I_{\mathrm{CaL}}$ inhibition [16], struggles to predict responses to milder $I_{\mathrm{CaL}}$ inhibition.

The TP model, though not validated against current reduction data, showed a low error measure (*E* = 76.6–83.8) but completely failed to reproduce the ${\text{APD}}_{90}$ increase induced by 100 nM Dofetilide ( + 26 ms predicted vs +256±21 ms experimentally). This is partially due to its significantly higher ${\text{I}}_{\text{Ks}}$ maximal conductance (0.392 mS/μF) compared to ORd-like models (from 0.0011 mS/μF in the ToR-ORd model to 0.0196 mS/μF in the ORd-M model), providing greater repolarisation reserve [21].

These findings highlight the importance of thoroughly examining model capabilities. This will help identifying in which context which AP model should (and should not) be used. The Cardiac Electrophysiology Web Lab facilitates this by testing models under various experimental protocols [22].

### 3.3. $\Delta {\text{APD}}_{90}$ in the context of proarrhythmic risk assessment

The TdP risk of 28 reference compounds was categorised under the CiPA initiative [7]. The ${\text{Q}}_{\text{net}}$ metric, simulated with the ORd-CiPA model, predicts TdP risk based on inhibition of major ionic currents [20]. The 2-D map for ${\text{Q}}_{\text{net}}$ (S1 Appendix), computed with similar methods to those for $\Delta {\text{APD}}_{90}$, shows that low TdP risk combinations of $I_{\mathrm{Kr}}$ and/or $I_{\mathrm{CaL}}$ inhibition qualitatively match the combinations leading to $\Delta {\text{APD}}_{\text{90}}\leq 0$ ms. This suggests a qualitative agreement between drug-induced $\Delta {\text{APD}}_{90}$ and TdP risk for drugs inhibiting $I_{\mathrm{Kr}}$ and $I_{\mathrm{CaL}}$.

Quinidine and Verapamil, which both inhibit similarly $I_{\mathrm{Kr}}$ and $I_{\mathrm{CaL}}$, exerted a mitigated effect on ${\text{APD}}_{90}$. Their $\Delta {\text{APD}}_{90}$ effects align with their effects on the ${\text{QT}}_{\text{c}}$ and ${\text{JT}}_{\text{peak}}$ interval of the ECG [9]. Our new *ex vivo* data suggest that sufficient $I_{\mathrm{CaL}}$ inhibition can prevent changes in ${\text{APD}}_{90}$, ${\text{QT}}_{\text{c}}$, and ${\text{JT}}_{\text{peak}}$ intervals, even at concentrations higher than $I_{\mathrm{Kr}}$ ${\text{IC}}_{50}$ — assuming the compound affects cardiomyocytes only through $I_{\mathrm{Kr}}$ and $I_{\mathrm{CaL}}$ inhibition. This may explain discrepancies between ICH S7B (high risk with $I_{\mathrm{Kr}}$ blockade) and ICH E14 (low risk with no ${\text{QT}}_{\text{c}}$ change) guidelines, potentially leading to false positives in pre-clinical risk assessments [23].

De Ponti estimated that 60% of new chemical entities inhibit $I_{\mathrm{Kr}}$, possibly including useful compounds with $I_{\mathrm{CaL}}$ inhibition mitigating the TdP risk [24]. But due to the prevalence of the $I_{\mathrm{Kr}}$–centric risk assessment, these compounds are rarely developed. Identifying combinations of $I_{\mathrm{Kr}}$ and $I_{\mathrm{CaL}}$ inhibition that do not prolong ${\text{APD}}_{90}$ could help develop compounds incorrectly deemed proarrhythmic. However, effects on blood pressure and myocardial contractility due to $I_{\mathrm{CaL}}$ inhibition still require attention.

### 3.4. Study limitations

The tested compounds were assumed to primarily affect $I_{\mathrm{Kr}}$ and $I_{\mathrm{CaL}}$, though literature suggests they may influence other ionic currents [20,25–29]. Moreover, the drug-binding kinetics may require more complex models than the simple Hill equation used here [30,31]. Ionic current response in adult cardiomyocytes may also differ from the response of hERG1a and ${\text{Ca}}_{\text{V}}1.2$ expression systems such as those used in the present work, for instance due to different native ion channels isoform and subunit composition or regulatory processes [32]. Our patch-clamp methods comply with the ICH S7B guideline [4] and best practices for *in vitro* assays, but refining these modelling assumptions with additional data would improve *in silico* predictions of drug responses.

The inhibitory potency of drugs on I_Kr_ and I_CaL_ differed between the CiPA and Pharm datasets, yet with a substantial correlation in ${\text{pIC}}_{50}$ across datasets (*r*^2^ = 0.84). No correlation was observed for *h* (*r*^2^ = 0.03). Incorporating this ${\text{IC}}_{50}$ variability when benchmarking models against *ex vivo* data enables a qualitative assessment of the models’ sensitivity to their *in vitro* inputs. In this study, ${\text{IC}}_{50}$ variability introduced substantial differences in $I_{\mathrm{Kr}}$ and $I_{\mathrm{CaL}}$ inhibition across datasets for some drug conditions (Fig 1). Yet, observations were overall consistent and the tested models showed consistent performance across both datasets (Fig 4). Individual variability was not addressed, but future work could incorporate a population of models approach [33] to account for it.

Most *ex vivo* data were generated from drugs with similar inhibitory effects on $I_{\mathrm{Kr}}$ and $I_{\mathrm{CaL}}$ (Chlorpromazine, Clozapine, Mesoridazine, Quinidine). These drugs mainly yielded small $\Delta {\text{APD}}_{90}$ responses, highlighting the importance of more detailed risk assessment for multi-channel ‘balanced’ inhibitors. Furthermore, our error measure, *E*, tends to favour models that accurately reproduce minimal ${\text{APD}}_{90}$ prolongation from mixed inhibition. An AP model predicting $\Delta {\text{APD}}_{\text{90}}=0$ ms for all combinations would score *E* = 59.0, outperforming all models studied here, indicating that *E* alone offers limited model comparison. Combining *E* with 2-D maps of $\Delta {\text{APD}}_{90}$ prediction (Fig 2) and error maps (Fig 3) helps identifying promising models for further refinement.

Concentration measurements were only available for half of the trabeculae, and drug concentrations were generally lower than nominal values, altering the positions of *ex vivo* $\Delta {\text{APD}}_{90}$ data points on our maps. Yet, the fitted cubic surface and model comparisons were not significantly impacted by the use of only nominal concentrations (S1 Appendix).

## 4. Methods

### 4.1. Ex vivo action potential acquisition

#### 4.1.1. Sharp electrode recording protocol for data acquisition.

Experimental AP data were produced by the AnaBios Corporation, following the methods previously described by Page *et al*. [34]. In brief, trabeculae were extracted from adult human hearts that were not suitable for transplantation, sharp electrodes were impaled in isolated cardiac muscle fibers, their electrophysiological activity was recorded at physiological temperature with vehicle or drugs added. Up to three trabeculae per heart were obtained from the inner endocardial wall of the left (78 trabeculae) and right (4 trabeculae) ventricles. 4 to 15 trabeculae were exposed to the same drug.

In each trabecula, the electrophysiological activity was recorded under baseline conditions, then with three increasing drug concentrations. After the last drug condition, a positive control for ${\text{APD}}_{90}$ prolongation with $I_{\mathrm{Kr}}$ inhibition was finally performed with 100 nM Dofetilide addition.

**Fig 5:**
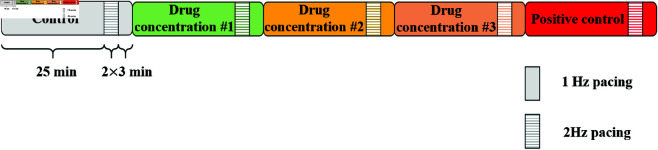
Protocol for sharp electrode recordings of the electrophysiological activity in isolated left- and right-ventricular human trabeculae. After baseline conditions, the response to three conditions with drug was recorded. At the end of the experiments, 100 nM Dofetilide was added as a positive control for ${\text{APD}}_{90}$ prolongation with $I_{\mathrm{Kr}}$ block.

At each drug concentration, each trabecula was paced at 1 Hz for a minimum of 25 min, until voltage recordings were stabilised for at least 2 mins. The stability of APs was assessed qualitatively by the experimenter, based on approximate measurements of the resting membrane potential (RMP), AP amplitude (APA) and ${\text{APD}}_{90}$. After reaching stable recordings, each trabecula was paced at 2 Hz for 3 min then paced again at 1 Hz for 3 min. The experimental protocol for drug administration is shown in Fig 5.

For more information on the experimental protocol, see [34].

#### 4.1.2. Selected drugs and tested drug concentrations.

The tested drugs inhibit $I_{\mathrm{Kr}}$ and $I_{\mathrm{CaL}}$ with various potencies, so that ${\text{APD}}_{90}$ changes induced by 29 different drug perturbations of $I_{\mathrm{Kr}}$ and $I_{\mathrm{CaL}}$ could be explored experimentally. The drugs used for this study and their concentrations are reported in Table 2. We call the intended drug concentration (targetted when making up solutions) the *nominal* concentration.

**Table 2:** Drugs tested in *ex vivo* experiments and corresponding nominal concentrations.

Drug	$1^{st}$ conc (μM)	$2^{nd}$ conc (μM)	$3^{rd}$ conc (μM)	$4^{th}$ conc (μM)	Number of trabeculae	Measured drug concentration
Chlorpromazine	0.3	1	3		6	Yes
Clozapine	0.3	1	3		7	Yes
Clozapine	0.3	3	30		4	No
Dofetilide	0.001	0.01	0.1	0.2	15	No
Fluoxetine	0.3	1	3		5	Yes
Mesoridazine	0.04	0.25	10		6	Yes
Nifedipine	0.003	0.03	0.3		4	No
Quinidine	0.1	1	10		15	No
Thioridazine	0.012	0.6	2		5	Yes
Verapamil	0.01	0.1	1		15	No

Experiments were undertaken in two distinct phases (2014–2016 and 2020–2022). In the second phase (2020–2022), a bioanalysis of the bath solution was performed to measure the drug concentration more precisely at the end of each 25 min period of steady 1 Hz pacing, in case the compound concentration was lowered by absorption by (e.g.) pipettes, tubing or the tissue itself. The sample analysis was performed according to the operating procedure for sample preparation for liquid chromatography–mass spectrometry or mass spectrometry analysis in a bioanalytical laboratory. For data gathered in the first phase (2014–2016), measured concentrations were not available, therefore drug concentrations were assumed to correspond to the nominal concentrations (Table 2).

#### 4.1.3. Ex vivo data post-processing.

Voltage was recorded with a time resolution of 0.05 ms and filtered to remove 60 Hz harmonics. The “peak voltage” for calculating percent repolarisation was measured as the upper 95th percentile of voltage [35]. The resting membrane potential (RMP) was the average voltage over the last 150 ms of the AP. ${\text{APD}}_{90}$ was computed from these reference points and averaged over 30 consecutive APs at the end of steady 1 Hz stimulation. $\Delta {\text{APD}}_{90}$ was defined as the difference from baseline ${\text{APD}}_{90}$. Recordings with 2 Hz pacing were not stabilised after 3 min, so $\Delta {\text{APD}}_{90}$ at 2 Hz was not analysed further.

Sudden changes in resting and peak voltages sometimes occurred, which were attributed to electrode movements: normalized APs showed these did not impact ${\text{APD}}_{90}$, so $\Delta {\text{APD}}_{90}$ was due to drug effects [36]. Data following voltage discontinuities were discarded if ${\text{APD}}_{90}$ was also suddenly altered. Conditions where early after-depolarisations (EADs) were observed (one trabecula with 100 nM Dofetilide) were not analyzed. $\Delta {\text{APD}}_{90}$ was finally averaged over trabeculae exposed to the same drug conditions.

Drug-induced $\Delta {\text{APD}}_{90}$ showed little correlation with baseline ${\text{APD}}_{90}$ (S1 Appendix). To align with clinical safety guidelines [5], $\Delta {\text{APD}}_{90}$ was not normalised to baseline and it therefore represents the absolute change from baseline.

### 4.2. Patch clamp measurements of $I_{\mathrm{Kr}}$ and $I_{\mathrm{CaL}}$ inhibition

Drug effects were modelled as simple pore block, using the Hill equation [37] to characterise it with a half-inhibitory concentration (${\text{IC}}_{50}$) and a Hill coefficient (*h*). Different voltage-clamp protocols were applied to CHO cells expressing hERG and ${\text{Ca}}_{\text{V}}1.2$ channels, and exposed to increasing drug concentrations to measure the drug-induced inhibition of ionic currents.

For $I_{\mathrm{Kr}}$ inhibition, two voltage-clamp protocols, denoted “$I_{\mathrm{Kr}}$ CiPA” [38] and “$I_{\mathrm{Kr}}$ Pharm”, were used. Similarly, two voltage-clamp protocols were used to measure the drug-induced $I_{\mathrm{CaL}}$ inhibition (“$I_{\mathrm{CaL}}$ CiPA” [20] and “$I_{\mathrm{CaL}}$ Pharm”). Thereby, two datasets for ${\text{IC}}_{50}$ and *h* for $I_{\mathrm{Kr}}$ and $I_{\mathrm{CaL}}$ were obtained, denoted the CiPA and Pharm datasets. With these two datasets, the impact of the variability of patch-clamp data [38] and of kinetics of drug binding to ion channels [39] can be qualitatively observed. For more details on the generation of the CiPA and Pharm datasets, please refer to S1 Appendix.

The retrieved ${\text{IC}}_{50}$ and *h* values are reported in Table 3. Dofetilide’s $I_{\mathrm{CaL}}$ ${\text{IC}}_{50}$ and Nifedipine’s $I_{\mathrm{Kr}}$ ${\text{IC}}_{50}$ were above the highest tested concentration. Therefore, Dofetilide was modelled as a selective $I_{\mathrm{Kr}}$ inhibitor and Nifedipine effect as a selective $I_{\mathrm{CaL}}$ inhibitor.

**Table 3:** Potency of inhibition of ${\text{Ca}}_{\text{V}}1.2$ and hERG channels for drugs tested *ex vivo*. The half-inhibitory concentration (${\text{IC}}_{50}$) is reported in microMolar (μM), and the Hill coefficient *h* is in brackets. “Pharm” and “CiPA” refer to two different patch-clamp protocols used to characterise $I_{\mathrm{Kr}}$ and $I_{\mathrm{CaL}}$ inhibition (S1 Appendix).

	$I_{\mathrm{CaL}}$ Pharm	$I_{\mathrm{CaL}}$ CiPA	$I_{\mathrm{Kr}}$ Pharm	$I_{\mathrm{Kr}}$ CiPA
**Drug**	${\text{IC}}_{50}$ (Hill)	${\text{IC}}_{50}$ (Hill)	${\text{IC}}_{50}$ (Hill)	${\text{IC}}_{50}$ (Hill)
Chlorpromazine	2.289 (0.88)	2.868 (0.93)	0.359 (0.84)	0.608 (0.69)
Clozapine	1.676 (0.752)	4.378 (0.932)	2.123 (1.05)	1.978 (0.94)
Dofetilide	>0.2	>0.2	0.029 (1.10)	0.033 (1.17)
Fluoxetine	0.994 (0.94)	0.857 (0.90)	0.712 (1.26)	0.772 (0.75)
Mesoridazine	4.056 (0.76)	3.962 (0.83)	0.503 (1)	0.565 (0.74)
Nifedipine	0.105 (0.85)	0.144 (0.72)	>8	>8
Quinidine	20.849 (0.63)	6.68 (1)	0.966 (1.01)	0.820 (1.43)
Thioridazine	0.497 (1.07)	0.637 (1.26)	0.171 (1)	0.154 (1.05)
Verapamil	1.381 (0.72)	0.310 (1)	0.273 (0.98)	0.570 (1.67)

### 4.3. Simulation of ${\text{APD}}_{90}$ with *in silico* action potential models

#### 4.3.1. Selected models.

We selected six main models representative of recent efforts to model the human ventricular AP: Ten Tusscher *et al*. (TNNP) [40], Ten Tusscher & Panfilov (TP) [41], Grandi *et al*. (GPB) [13], O’Hara *et al*. (ORd) [12], Tomek *et al*. (ToR-ORd) [14], and Bartolucci *et al*. (BPS) [16]. Since their release, five new parameterisations and variants of these models have been published. Dutta *et al*. replaced the $I_{\mathrm{Kr}}$ component of the ORd model with a 6-state Markov model [7] and rescaled the maximal conductances of five ionic currents ($I_{\mathrm{Kr}}$, $I_{\mathrm{CaL}}$, ${\text{I}}_{\text{K1}}$, ${\text{I}}_{\text{Ks}}$, ${\text{I}}_{\text{NaL}}$) [42]. The GPB, TP, and ORd models were rescaled by Mann *et al*. to capture the effects of $I_{\mathrm{Kr}}$ and ${\text{I}}_{\text{Ks}}$ inhibition and to reproduce ${\text{APD}}_{90}$ features observed in long QT Syndrome (LQTS) populations [11]. Mann *et al*. added a late sodium component to their versions of the GPB and TP models, based on the ORd model. Krogh-Madsen *et al*. proposed a version of the ORd model with rescaled maximal conductance parameters for six ionic currents ($I_{\mathrm{Kr}}$, $I_{\mathrm{CaL}}$, ${\text{I}}_{\text{Ks}}$, ${\text{I}}_{\text{NaCa}}$, ${\text{I}}_{\text{NaK}}$, ${\text{I}}_{\text{NaL}}$) to capture populations with long QT syndrome, which was also included in the present study [15]. All these variant models were included in the present study and are summarised in Table 4.

**Table 4:** Selected AP models. The ^*^ symbol indicates when the endocardial version of the model was selected among different versions developed by the authors of the model.

Model	Reference	Model structure
${\text{BPS}}^{*}$	[16]	ORd + $I_{\mathrm{CaL}}$
${\text{GPB10}}^{*}$	[13]	GPB
GPB-M	[11]	GPB + ORd ${\text{I}}_{\text{NaL}}$
${\text{ORd}}^{*}$	[12]	ORd
ORd-${\text{CiPA}}^{*}$	[42]	ORd + $I_{\mathrm{Kr}}$
ORd-KM	[15]	ORd
ORd-M	[11]	ORd
ToR-${\text{ORd20}}^{*}$	[14]	ToR-ORd
TNNP	[40]	TNNP
${\text{TP}}^{*}$	[41]	TP
TP-M	[11]	TP + ORd ${\text{I}}_{\text{NaL}}$

To maximise consistency with the trabeculae measurements, the endocardial variant of the AP models was used when available.

#### 4.3.2. Action potential simulations.

Published CellML models were obtained from the Physiome Repository [43]. Stimulus current width, amplitude, and responsible ions (for instance ${\text{K}}^{+}$ [44]) were not changed. 1 Hz steady pacing was applied in line with the *ex vivo* experiments. We simulated 1500 s to reach a steady-state response to 1 Hz pacing. In all models, the convergence to steady-state was achieved with 1500 pre-paces. The $1501^{\text{st}}$ AP was then recorded with time resolution of 0.05 ms, matching the resolution of the *ex vivo* data, and allowing for precise estimation of ${\text{APD}}_{90}$.

When computing 2-D maps of $\Delta {\text{APD}}_{90}$ as a function of $I_{\mathrm{Kr}}$ and $I_{\mathrm{CaL}}$ inhibition (Sect 2.2), default initial internal and external concentrations from the CellML files were used. When comparing quantitative model predictions with trabeculae recordings (Sect 2.3), external concentrations of ${\text{K}}^{+}$, ${\text{Na}}^{+}$, and ${\text{Ca}}^{2+}$ were set to 4 mM, 148.35 mM, and 1.8 mM respectively, matching concentrations used experimentally [34].

**Fig 6:**
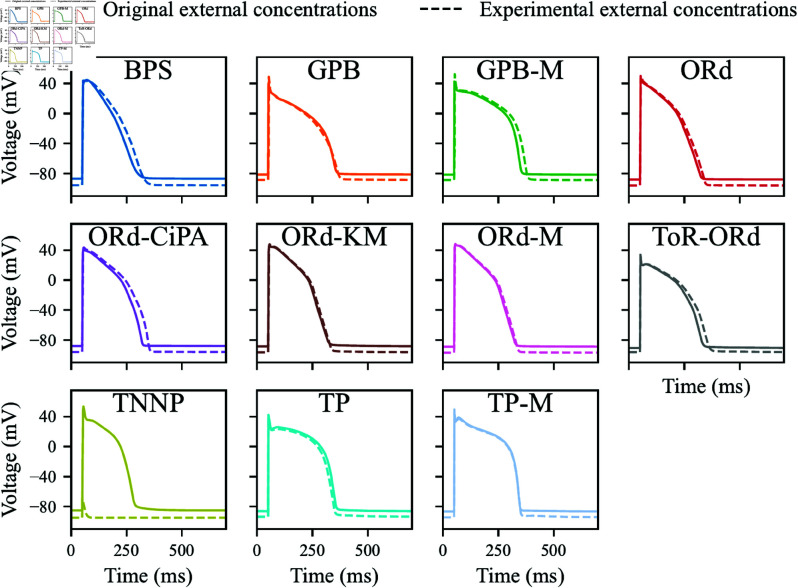
Steady-state 1 Hz AP simulated with the AP models included in this study. External concentrations were set to experimental values (**dashed line**) or left at the values in the original CellML model (**solid line**).

The steady-state APs simulated with the included models are shown in Fig 6 for visual comparison. Note that the TNNP model does not predict a physiological AP when external ionic concentrations match experimental values. Therefore, predictions with the TNNP model were not quantitatively compared with the *ex vivo* data in Sect 2.3.

*In vitro* data for inhibition of $I_{\mathrm{Kr}}$ and $I_{\mathrm{CaL}}$ were integrated into model predictions by applying a rescaling factor, computed with the Hill equation [37,45], to each affected ionic current:

$$I(D)=\frac{1}{1+{\left(\frac{D}{{\text{IC}}_{50}}\right)}^{h}}\times I_{0},$$ (1)

with *I*(*D*) the simulated ionic current, *D* the drug concentration, and *I*_0_ = *I*(0) the ionic current without drug. ${\text{IC}}_{50}$ and *h* were taken from Table 3.

### 4.4. Comparison of model predictive power with experimental action potential data

#### 4.4.1. Qualitative comparison with 2-D maps of $\Delta {\text{APD}}_{90}$ versus current inhibition.

With each model, we simulated APs under 101×101=10,201 combinations of $I_{\mathrm{Kr}}$ and $I_{\mathrm{CaL}}$ inhibition conditions, ranging from 0% to 100% inhibition. $\Delta {\text{APD}}_{90}$ was computed for each $I_{\mathrm{Kr}}$/$I_{\mathrm{CaL}}$ inhibition combination. $\Delta {\text{APD}}_{90}$ was shown using a colour-map that was kept consistent across all the models and which covered the experimental range of drug-induced $\Delta {\text{APD}}_{90}$. Combinations of $I_{\mathrm{Kr}}$ and/or $I_{\mathrm{CaL}}$ inhibition for which no change in ${\text{APD}}_{90}$ were observed or predicted ($\|\Delta {\text{APD}}_{90}\|⩽1$ ms) were plotted as white pixels, thus highlighting a “0 ms line”.

The experimental drug-induced $\Delta {\text{APD}}_{90}$ data was reported with similar methods. Additionally, a cubic surface of $\Delta {\text{APD}}_{90}$ as a function of $I_{\mathrm{Kr}}$ and $I_{\mathrm{CaL}}$ inhibition was fitted through the *ex vivo* data points (details in S1 Appendix) to facilitate visual comparison of *ex vivo* data with simulations.

**Fig 7:**
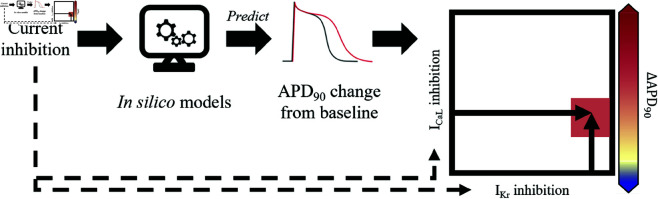
Schematic of methods used to plot $\Delta {\text{APD}}_{90}$ 2-D maps. Simulated $\Delta {\text{APD}}_{90}$ was computed from the *in silico* AP model run for 1500 paces, using $I_{\mathrm{Kr}}$ and/or $I_{\mathrm{CaL}}$ inhibition as input for the model. The corresponding point was then added to the 2-D map, with $\Delta {\text{APD}}_{90}$ reported with the colour-map.

Fig 7 shows a schematic visualisation of the methods for 2-D map simulations.

#### 4.4.2. Metric for the quantification of model predictivity.

To quantitatively compare $\Delta {\text{APD}}_{90}$ measurements and predictions, APs were simulated at all nominal drug concentrations, or at actual drug concentrations where available. An error measure, *E*, was then designed to quantify the error in predicted $\Delta {\text{APD}}_{90}$ whilst accounting for experimental variability. *E* was defined as:

$$E=\sum_{k}^{K}\left|\frac{\Delta {\text{APD}}_{90,\text{sim,k}}-\overset{―}{\Delta }{\text{APD}}_{90,\text{exp,k}}}{\sigma_{\text{M,exp},k}}\right|,$$ (2)

with $\overset{―}{\Delta }{\text{APD}}_{90,\text{exp,k}}$ and $\Delta {\text{APD}}_{90,\text{sim,k}}$ the experimental and simulated $\Delta {\text{APD}}_{90}$ for the drug perturbation *k*, and $\sigma_{\text{M,exp},k}$ the SEM of experimental $\Delta {\text{APD}}_{90}$ across the trabeculae tested with *k*. Indices *k* span all concentrations of the nine drugs (Table 2).

## 5. Conclusion

Our new experimental data provide quantitative understanding of the relationship between ${\text{APD}}_{90}$ and acute $I_{\mathrm{Kr}}$ and/or $I_{\mathrm{CaL}}$ inhibition in adult human ventricular cardiac muscle. Combined with *in vitro* data, they make a valuable benchmark for assessing the performance of *in silico* AP models. Although certain models accurately predict ${\text{APD}}_{90}$ prolongation for selective $I_{\mathrm{Kr}}$ inhibitors, they struggle to replicate the mitigating effects of simultaneous $I_{\mathrm{CaL}}$ inhibition observed experimentally. The TP and GPB-M models appear to be the most promising starting points for developing more advanced AP models that can account for multi-ion channel inhibition in cardiac safety risk predictions. Of the ORd-like models, the ToR-ORd model exhibits the most promising balance between $I_{\mathrm{Kr}}$ and $I_{\mathrm{CaL}}$ and its predictivity may be improved upon reparameterisation. Our study emphasises the importance of context-specific validation of AP models: rigorous testing across various ion channel inhibitions and drug concentrations is essential to ensure models can reliably predict cardiac responses. Extended model validation, alongside high-quality experimental data, will ultimately lead to more accurate and reliable *in silico* frameworks for predicting proarrhythmic risk from *in vitro* data. Such frameworks can be vital in improving the specificity of early identification of potential cardiac safety issues in drug development.

## Declaration of generative AI and AI-assisted technologies in the writing process

During the preparation of this work the authors used ChatGPT (public v3 and internal Roche v4O) in order to enhance general text readability and conciseness, and to debug code. After using this tool, the authors reviewed and edited the content as needed and take full accountability for the content of the publication.

## Supporting information

S1 AppendixAll supplementary materials, including Supplementary Figs A–K and Tables A–E.The appendix includes details on patch-clamp experimental protocols, additional figures supporting the main text, and a 2-D map of ${\text{Q}}_{\text{net}}$.(PDF)
