# Supplementary material for: Comparison of in silico predictions of action potential duration in response to inhibition of IKr and ICaL with new human ex vivo recordings
Source: PLoS Comput Biol. 2025 Jul 7;21(7):e1012913. doi: 10.1371/journal.pcbi.1012913 (PMC12251226; doi:10.1371/journal.pcbi.1012913)
Supplement: S1 Appendix — The appendix includes details on patch-clamp experimental protocols, additional figures supporting the main text, and a 2-D map of Qnet. (PDF) [file pcbi.1012913.s001.pdf]

Appendix: Comparison of in silico predictions of action potential duration in response to inhibition of I_{Kr} and I_{CaL} with new human ex vivo recordings

April 29, 2025

List of Figures

A Protocols for recording of the peak $I_{CaV1.2}$ current. 4

B Protocols for recording of the peak I_{hERG} current. 5

C Error in the cubic surface compared with the experimental ΔAPD_{90} data 8

D Impact of reporting drug concentrations as measured in the bath solution or as nominal 16

E Comparison of the abilities of human ventricular AP models to reproduce the APD_{90} response to I_{Kr} and I_{CaL} inhibition observed *ex vivo*, using only nominal drug concentrations as inputs for simulations 17

F Correlation between baseline APD_{90} and Verapamil-induced perturbations 18

G Experimental $\%\Delta APD_{90}$ measured ex-vivo under various drug conditions in human ventricular trabeculae 18

H 2-D maps of simulated $\%\Delta APD_{90}$after I_{CaL} and I_{Kr} inhibition 19

I Observed versus prediction plot for the tested models, using the CiPA and Pharm datasets 20

J Q_{net} computed with the ORd-CiPA model, for various combinations of I_{Kr} and/or I_{CaL} inhibition 21
K Stability of AP markers observed in a representative trabecula 22

List of Tables

A Data summary of IC_{50} values for hERG and $Ca_V1.2$ block potency of the Clozapine. 6
B Data summary of IC_{50} values for hERG and $Ca_V1.2$ block potency of Dofetilide. 6
C Data summary of IC_{50} values for hERG and $Ca_V1.2$ block potency of Nifedipine. 7
D Data summary of IC_{50} values for hERG and $Ca_V1.2$ block potency of Quinidine. 7
E Data summary of IC_{50} values for hERG and $Ca_V1.2$ block potency of Verapamil. 8

1 In vitro measurements of I_{Kr} and I_{CaL} inhibition

For a consistent comparison of the APD_{90} response to drug perturbation predicted by the AP models included in the present benchmark, in some of which the dynamic hERG binding model cannot be implemented, the inhibition of ionic currents was modelled with the Hill equation:

$$I(D) = \frac{1}{1 + \left(\frac{D}{\mathrm{IC}_{50}}\right)^h} \times I(0), \tag{1}$$

with I the current with drug inhibition, D the drug concentration, h the Hill coefficient, IC_{50} the half inhibitory drug concentration, and $I(0)$ the ionic current measured at baseline without any drug exposure.

The AP models considered in this study do not distinguish isoforms of the ion channels, thus the drug inhibition of hERG and $Ca_V1.2$ channels was modelled as equal to the drug inhibition on I_{Kr} and I_{CaL}, respectively. hERG and $Ca_V1.2$ channels are the main ion

channels responsible for I_{Kr} and I_{CaL}, respectively (Agrawal *et al.*, 2022; Sanguinetti *et al.*, 1995; Li *et al.*, 1996).

The experiments were carried out internally in Roche by Evgenia Gissinger and Fabian Häusermann, from Dr. Liudmila Polonchuk's lab (Roche Pharmaceutical Research and Early Development, Pharmaceutical Sciences).

1.1 Cell culture

The CHO crelox hERG cell line was generated and validated at Roche (Guthrie *et al.*, 2005). The CHO-hCa_V1.2/β2/α2δ cell line was purchased from ChanTest (USA, Catalog #CT6004). Vials with cryopreserved cells were thawed at 37°C, washed with the pre-warmed IMDM cell culture medium (Gibco Life Technologies, USA) and re-suspended in the extracellular solution.

For the hERG assay the extracellular solution contained (in mM): NaCl 80; KCl 4; $CaCl_2$ 1; $MgCl_2$ 1; NMDG 40; HEPES 10; sorbitol 40; glucose 5; pH 7.2–7.4 with NaOH, osmolarity 290-330 mOsm and the internal solution contained (in mM): KCl, 10; KF, 100; NaCl, 10; HEPES, 10; EGTA, 20; pH = 7.0–7.4 with KOH, osmolarity 260-300 mOsm. For the L-type Ca_V1.2 assay the extracellular solution contained (in mM): NaCl 80; KCl 4; $CaCl_2$ 1.8; $MgCl_2$ 1; NMDG 40; HEPES 10; sorbitol 40; glucose 5; pH 7.2–7.4 with NaOH, osmolarity 290-330 mOsm and the internal solution contained (in mM): KCl, 10; KF, 100; NaCl, 5; HEPES, 10; EGTA, 10; Na-ATP, 4; Na-GTP, 0.1; pH = 7.0–7.4 with KOH, osmolarity 260–300 mOsm.

1.2 Electrophysiology recordings

The recording of the currents was performed using automated patch clamp system SynchroPatch 384 (Nanion Technologies GmbH, Germany) at 35–37°C following the experimental procedure described below. On the day of the experiment, an aliquot of the cell suspension in a 2:1 mixture of the HBSS and external solution was placed in the Cellhotel. The cells were subsequently added into the 384-well sealchip where the currents were recorded in single cells with the patch-voltage-clamp technique in the whole-cell

configuration at 35–37°C using the built-in 384 channel amplifier and associated software (PatchControl 384). Currents were low-pass filtered using the analog 3 kHz Bessel filter and the digital 3 kHz Lanczos filter and were digitized at 5 kHz. Series resistance was typically 2–9 MΩ and is compensated by 80%. The reported current amplitudes represent the maximal amplitude of a peak current.

1.3 Voltage-clamp protocols

The voltage-step protocols used to measure the $Ca_V1.2$ inhibition by drugs are shown in Figure A. In the Roche in-house protocol (Pharm dataset), the cells were held at a resting potential of −90 mV, where the baseline current amplitude was recorded. The $Ca_V1.2$ channels were then activated with 120 ms-wide steps at 0 mV with a frequency of 0.1 Hz, where the peak current was recorded. Once the recorded peak and baseline currents were stabilised, the amplitude and kinetics of the $I_{CaV1.2}$ were recorded for 3–5 minutes without drug. Then, after each drug addition, the activity of the cells was recorded during 3 minutes.

In the CiPA protocol, cells were held at a resting potential of −80 mV, where the baseline current was recorded. Then a stimulus 40 ms step of voltage at 0 mV was applied to record the peak current, with a 0.1 Hz frequency. Then the voltage was held at +30 mV for 200 ms, followed by a ramp down of voltage to return to −80 mV in 99 ms.

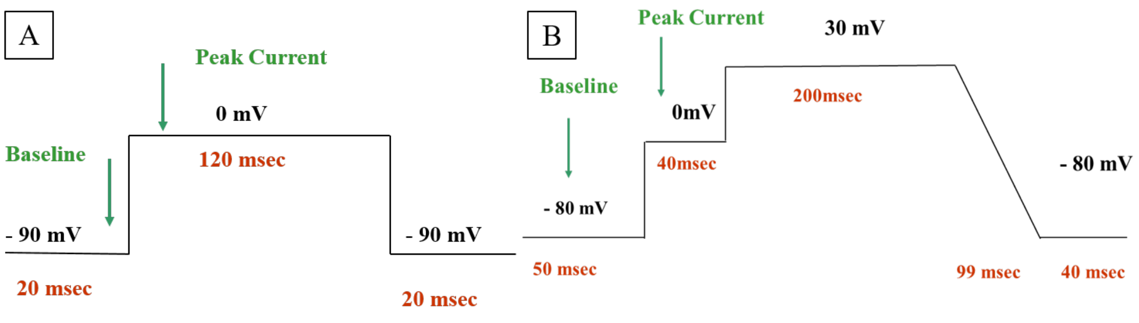

Figure A: Protocols for recording of the peak $I_{CaV1.2}$ current. **A:** Roche in-house protocol (‘Pharm’ dataset). **B:** CiPA protocol (‘CiPA’ dataset) (Li *et al.*, 2019).

The voltage-step protocols used to measure the hERG inhibition by drugs are shown in Figure B. In the Roche in-house protocol, the resting voltage was set to −80 mV. Then

the voltage was clamped to $-40\,\text{mV}$ for $100\,\text{ms}$, where the baseline current was recorded. The voltage was then brought to $+20\,\text{mV}$ for $500\,\text{ms}$ and finally to $-40\,\text{mV}$ for $500\,\text{ms}$, where the peak current was recorded. Afterwards, the voltage was set back to the resting potential of $-80\,\text{mV}$. The stimulation pattern is repeated with a frequency of $0.1\,\text{Hz}$.

In the CiPA protocol, the cells were held to the resting potential of $-80\,\text{mV}$. A $+40\,\text{mV}$ voltage was then applied to them for $500\,\text{ms}$, followed by a $-1.25\,\text{mV/ms}$ ramp that brought the voltage down to the resting potential in $96\,\text{ms}$. The peak current was recorded during this ramp down. The pattern was repeated with a frequency of $0.1\,\text{Hz}$.

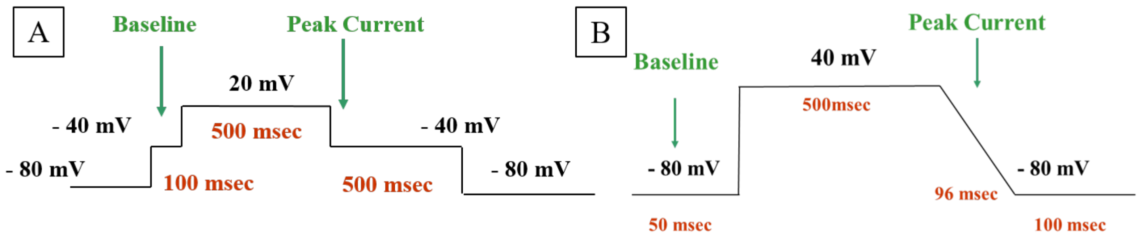

Figure B: Protocols for recording of the peak I_{hERG} current. **A:** Roche in-house (‘Pharm’ dataset). **B:** CiPA protocol (‘CiPA’ dataset).

1.4 Literature and new IC50 data

The Tables A to E below summarise the IC_{50} values that could be found in the literature for hERG and $Ca_V1.2$ block potency of Clozapine, Dofetilide, Nifedipine, Quinidine and Verapamil. Overall, there was good agreement between literature values and the values we present in this study. Nifedipine IC_{50} (measured with both protocols) was however higher than literature values for $Ca_V1.2$. The 1.5 inter-quartile range statistical test was performed to identify outliers, and no outlier was found.

Drug	hERG block IC50 (μM)/h	Ca_V1.2 block IC50 (μM)/h	Source
Clozapine	1.978 / 0.94	4.378 / 0.93	CiPA prot.
	2.123 / 1.05	1.676 / 0.75	Pharm prot.
	2.300 / 0.97	3.600 / 1	(Kramer *et al.*, 2013)
		5.490 / 0.94	(Li *et al.*, 2019)
	2.310 / 1	3.555 / 1	One Million Solutions in Health
	1.639 / 1	3.600 / 1	(Llopis-Lorente *et al.*, 2020)

Table A: Data summary of IC_{50} values for hERG and Ca_V1.2 block potency of the Clozapine.

Drug	hERG block IC50 (μM)/h	Ca_V1.2 block IC50 (μM)/h	Source
Dofetilide	0.033 / 1.17		CiPA prot.
	0.029 / 1.10	332 / 1	Pharm prot.
	0.030 / 1.2	26.7 / 1	(Kramer *et al.*, 2013)
	0.0061 / 1.08	44.5 / 3.6	CiPA GitHub
	0.005 / 1	60 / 1	(Mirams *et al.*, 2011)
	0.002 / 1		(Redfern *et al.*, 2003)
	0.002 / 1		(Crumb Jr *et al.*, 2016)
	0.037 / 2.1	182 / 1	(Okada *et al.*, 2015)
	0.025 / 1	26.7 / 1	(Llopis-Lorente *et al.*, 2020)

Table B: Data summary of IC_{50} values for hERG and Ca_V1.2 block potency of Dofetilide.

2 Fitting of the cubic surface through the experimental ΔAPD_{90} data

Cubic surfaces were fitted through the experimental data for drug-induced ΔAPD_{90}. The cubic surface was computed following the equation:

$$z = \text{surf}(x, y, \boldsymbol{\theta}) = \theta_1 x^2 + \theta_2 y^2 + \theta_3 xy + \theta_4 x + \theta_5 y + \theta_6 x^3 + \theta_7 y^3 + \theta_8 x^2 y + \theta_9 xy^2, \quad (2)$$

with z the approximated ΔAPD_{90}, x and y the percentage of inhibition of I_{Kr} and I_{CaL}, respectively, and $\boldsymbol{\theta}$ the parameters describing the cubic surface. The location of each data point for each tested trabecula (x and y) was computed using the IC50 data (Table 2 in the main text) and the measured drug concentration in the bath solution if available. The

**Drug**	**hERG block IC50 (μM)/h**	**Ca$_V$1.2 block IC50 (μM)/h**	**Source**
Nifedipine	inactive	0.144 / 0.72	CiPA prot.
	inactive	0.105 / 0.85	Pharm prot.
		0.0114 / 0.67	(Li *et al.*, 2019)
	44 / 0.8	0.012 / 1.02	(Kramer *et al.*, 2013)
	275 / 1	0.060 / 1	(Mirams *et al.*, 2011)
		0.056 / 1.18	(Elkins *et al.*, 2013)
	112.25 / 1	0.060 / 1	(Llopis-Lorente *et al.*, 2020)
		0.016 / 1	Kuryshev *et al.* (2014)

Table C: Data summary of IC_{50} values for hERG and Ca$_V$1.2 block potency of Nifedipine.

**Drug**	**hERG block IC50 (μM)/h**	**Ca$_V$1.2 block IC50 (μM)/h**	**Source**
Quinidine	0.820 / 1.43	6.680 / 1	CiPA prot.
	0.966 / 1.01	20.849 / 0.63	Pharm prot.
	2.371 / 1.71		(Elkins *et al.*, 2013)
	0.3 / 1	15.6 / 1	(Mirams *et al.*, 2011)
	0.72 / 1.06	6.4 / 0.68	(Kramer *et al.*, 2013)
	0.3 / 1		(Crumb Jr *et al.*, 2016)
	0.658 / 1	8.1 / 0.8	(Okada *et al.*, 2015)
	0.986 / 0.84	51.59 / 0.59	CiPA GitHub
	0.890 / 1	8.866 / 0.912	(Llopis-Lorente *et al.*, 2020)

Table D: Data summary of IC_{50} values for hERG and Ca$_V$1.2 block potency of Quinidine.

nominal drug concentration was used otherwise.

The cubic surface was fitted through the experimental ΔAPD_{90} data points, by minimising the cost function:

$$\mathcal{S}(\boldsymbol{\theta}) = \sum_{\text{trab}} \sum_{k} \left(\text{surf}(x_k, y_k, \boldsymbol{\theta}) - \Delta \text{APD}_{90,\text{exp,k, trab}}\right)^2, \quad (3)$$

with $\Delta APD_{90,\text{ exp, k, trab}}$ the experimental ΔAPD_{90} for the drug perturbation k averaged over 30 consecutive APs in the trabecula *trab*. For each k, the associated inhibition of I_{Kr} and I_{CaL} was computed using Eq. 2 and the IC50 data reported in Table 2 in the main text. The minimisation of the cost function was performed using the `scipy` Python package. Based on experimental observations, the following constraints were put on the cubic surface during its fitting:

Drug	hERG block IC50 (μM)/h	$Ca_V1.2$ block IC50 (μM)/h	Source
Verapamil	0.570 / 1.67	0.310 / 1	CiPA prot.
	0.273 / 0.98	1.381 / 0.72	Pharm prot.
	0.296 / 0.94	0.202 / 1.10	CiPA GitHub
	0.250 / 0.89	0.200 / 0.8	(Kramer *et al.*, 2013)
	0.677 / 1.43	2.685 / 0.61	(Elkins *et al.*, 2013)
	0.143 / 1	0.100 / 1	(Mirams *et al.*, 2011)
	0.7 / 1	0.1 / 1	(Crumb Jr *et al.*, 2016)
	0.212 / 1	0.347 / 1.08	(Okada *et al.*, 2015)
	0.499 / 1.1	0.201 / 1.1	(Llopis-Lorente *et al.*, 2020)

Table E: Data summary of IC_{50} values for hERG and $Ca_V1.2$ block potency of Verapamil.

- $\Delta APD_{90} = 0$ ms at baseline, i.e., $\theta_6 = 0$;
- $\Delta APD_{90} \leq -50$ ms for 100% I_{CaL} block ;
- $\Delta APD_{90} \geq +320$ ms for 100% I_{Kr} block ;
- $\frac{dz}{dx} > 0$, translating that an increase in I_{Kr} inhibition prolongs the APD_{90};
- $\frac{dz}{dy} < 0$, translating that an increase in I_{CaL} inhibition shortens the APD_{90}.

When the constraints were not satisfied, the cost (Eq. 3) was multiplied by 100.

The goodness of fit of the cubic surface to the experimental points is plotted in Figure C.

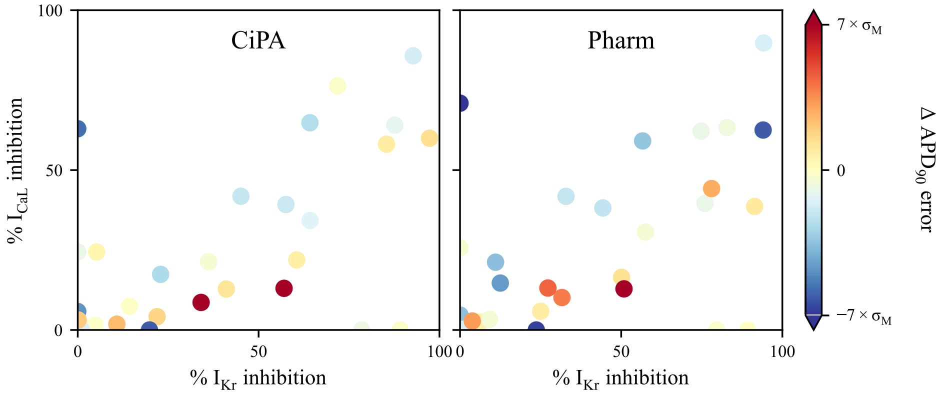

Figure C: Error (Eq. 2) in the cubic surface compared with the experimental ΔAPD_{90} data, obtained using the CiPA (**left**) and the Pharm dataset (**right**).

3 Comparison of 2D maps using nominal concentrations and using measured drug concentrations in the bath solution (when available)

As reported in Table 1, drug concentrations in the bath solution were measured for some compounds, while they were not measured for other compounds. Note that for Clozapine, the drug concentration was measured in 7 trabeculae exposed to 0.3–3 μM but not in the 4 trabeculae exposed to 0.3–30 μM. In this section we compare whether fitting the cubic surface to nominal or measured drug concentrations makes any visual change to the resulting surface, and if simulating the drug-induced ΔAPD_{90} with nominal concentrations impacts our results.

The fitting of the cubic surface was repeated three times, using differently the data for drug concentration to locate the experimental data points on the 2-D map (Eq. 2):

- using the measured drug concentration in the bath solution for each trabecula under each tested drug condition, when the drug concentration was measured. Otherwise, the nominal concentration was used. In this case, ΔAPD_{90} is reported separately for each trabecula for each drug condition ;
- averaging the measured drug concentration for all trabeculae tested with the same nominal drug concentration, when the drug concentration was measured. Otherwise, the nominal drug concentration was used. The drug-induced ΔAPD_{90} effect was averaged over the trabeculae tested with the same nominal concentration ;
- using only the nominal drug concentrations. The drug-induced ΔAPD_{90} effect was averaged over the trabeculae tested with the same nominal concentration.

The experimental data points were placed on the 2-D map following the three methods, and the corresponding cubic surfaces were fitted through these data points. The fitting of the cubic surface was repeated with the CiPA and Pharm protocols, and the results are visualised in Figure D.

The exact location of points was changed due to the measured concentrations in the bath solution not matching exactly with the nominal concentrations. Yet, the cubic surfaces fitted to the experimental points with the three methods described above were very similar. Therefore, the qualitative comparison of model predictions with the experimental data yields the same results when the nominal drug concentrations are used to compute the drug-induced inhibition of I_{Kr} and I_{CaL}.

To further support that the interpretation of our results were not sensitive to the discrepancy between measured drug concentrations in the bath solution and nominal drug concentrations, the scores were recomputed with all nominal concentrations, similarly to Figure 7. The results, plotted in Figure E, are similar to the results obtained with measured drug concentrations in the bath solution (when available), and the interpretation of our results therefore did not depend on the discrepancy between the measured and nominal drug concentrations.

4 Comparison of drug-induced ΔAPD_{90} with relative APD_{90} change from baseline as a percentage

To investigate whether drug-induced changes in APD_{90} should be reported in absolute or relative values, the correlation between baseline APD_{90} and response to drug perturbation was observed. For 15 trabeculae exposed to 1 μM Verapamil, the absolute change in APD_{90} (ΔAPD_{90}) was computed. From there, the relative change in APD_{90} was computed with the following equation:

$$\%\Delta APD_{90} = \Delta APD_{90}/APD_{90,\text{baseline}}, \tag{4}$$

$APD_{90,\text{ baseline}}$ referring to the baseline APD_{90}.

The scatter plot of ΔAPD_{90} and $\%\Delta APD_{90}$ against baseline APD_{90} is plotted in Figure F. The ΔAPD_{90} and $\%\Delta APD_{90}$ showed not correlated with baseline APD_{90}. Consistent results were obtained for all drugs and all drug concentrations.

Therefore, normalising ΔAPD_{90} would not improve the understanding of drug effect. Furthermore, in the clinic, changes in QT are measured in absolute, average prolongation of the QT interval by more than +5 ms being the limit of tolerance (ICH, 2006). At the cellular level, ΔAPD_{90} is more directly linked with the safety marker than $\%\Delta APD_{90}$. As a conclusion, the ΔAPD_{90} was used for the rest of this study.

To investigate further the importance of using ΔAPD_{90} over $\%\Delta APD_{90}$ in this study the results of the main text were repeated using the relative APD_{90} change expressed as a percentage of the baseline APD_{90} ($\%\Delta APD_{90}$), instead of ΔAPD_{90}. The relative APD_{90} change was computed in each trabecula as:

$$\Delta_{\%}APD_{90} = 100 \times \Delta APD_{90}/APD_{90,\text{ baseline}}. \tag{5}$$

The experimental $\%\Delta APD_{90}$is plotted in Figure G. The cubic surface was similar to the cubic surface observed in Figure 4, granted the scalings differed between the two figures.

Predictions of $\%\Delta APD_{90}$ were also computed for the 11 AP models, and plotted in Figure H. Very similar trends were observed in 2-D maps for ΔAPD_{90} and $\%\Delta APD_{90}$. Therefore, the choice of using ΔAPD_{90} over $\%\Delta APD_{90}$ did not impact the interpretation of the results presented in this study.

5 Model predictions vs observations

Model predictions are plotted against experimental observations in Figure I. It enables an extra visualisation of the model performances, similar to Figure 6 in the main text. The same observations can be made as described in the main text. The ORd-like models capture the strong APD_{90}-prolonging perturbations, but they largely overpredict the APD_{90} response to compounds inhibiting both I_{Kr} and I_{CaL}. The TP-like models are less prone to overprediction, but they are unable to predict strong APD_{90} prolongation.

6 The TdP risk metric Q_{net} as a function of I_{Kr} and I_{CaL} inhibition

The CiPA initiative was established with the objective of developing an *in silico* model classifying drugs into three TdP risk categories (low, intermediate, high risk), providing a more specific safety assessment than the I_{Kr}-centric guideline (Sager *et al.*, 2014). One popular candidate, Q_{net}, relies on the net charge flux over the repolarisation phase of one AP computed with the ORd-CiPA model (Li *et al.*, 2019). Q_{net} is defined as the integral of the net currents that are active in the repolarisation phase over one AP at 0.5 Hz pacing after 1000 pre-paces defined as:

$$Q_{net} = \int_{0\,\mathrm{ms}}^{2000\,\mathrm{ms}} \left(I_{Kr} + I_{CaL} + I_{Ks} + I_{NaL} + I_{K1} + I_{to}\right) \mathrm{d}t. \tag{6}$$

In this section, we compare Q_{net} as a function of I_{Kr} and I_{CaL} inhibition with experimental ΔAPD_{90} measurements.

As with the AP models, Q_{net} was computed with the ORd-CiPA model for $101 \times 101 = 10,201$ combinations of I_{Kr} and I_{CaL} inhibition. The reduction of I_{Kr} was modelled by applying a multiplying factor to the maximal conductance of I_{Kr}, as in the main text, although in the original methods of Li *et al.*, the I_{Kr} inhibition by drugs is modelled with the dynamic hERG binding model (Li *et al.*, 2017). Note that Li *et al.* classified compounds into the TdP risk categories based on the average Q_{net} computed at 1–4 times their maximal effective free therapeutic concentration. Nevertheless, a qualitative interpretation of the 2-D map remains possible.

Pixels of the 2-D map were colored based on the TdP risk category corresponding to Q_{net} obtained with the ORd-CiPA model after inhibition of the ionic currents. As in (Li *et al.*, 2019), Q_{net} values greater than $0.0671\,\mu C.\mu F^{-1}$ were classified as low risk (green), Q_{net} between $0.0581\,\mu C.\mu F^{-1}$ and $0.0671\,\mu C.\mu F^{-1}$ as intermediate risk (blue), and Q_{net} lower than $0.0581\,\mu C.\mu F^{-1}$ as high risk.

The resulting 2-D map is shown in Figure J.

Interestingly, the decrease in Q_{net} (increase in TdP risk) induced by I_{Kr} inhibition is

mitigated by I_{CaL} inhibition, with a higher sensitivity to I_{CaL} inhibition than ΔAPD_{90} predicted by the ORd-CiPA model. For example, 50% I_{Kr} inhibition and 75% I_{CaL} inhibition yields a Q_{net} value classified into the low TdP risk category, while the predicted ΔAPD_{90} is +73 ms.

The shape of the 2-D map of Q_{net} is similar to that of the 2-D map of ΔAPD_{90} predicted by the TP-M model (Figure 5 in the main text). Furthermore, qualitatively similar mitigation of I_{Kr} inhibition by I_{CaL} inhibition was observed between Q_{net} predictions and ΔAPD_{90} observed experimentally (Figure 4 in the main text).

7 Stability of AP markers after 25 minutes of steady pacing

In *ex vivo* experiments, a steady 1 Hz pacing was applied to trabeculae (Figure 1). The stability of the AP was qualitatively assessed by the experimenter, based on APD_{90}, AP amplitude, and RMP. If stability was not achieved after 25 min, the steady pacing was prolonged. The evolution of these AP markers is plotted in Figure K for a representative trabecula, for the last 2 min of steady pacing after stabilisation.

References

A Agrawal, K Wang, L Polonchuk, J Cooper, M Hendrix, DJ Gavaghan, GR Mirams and M Clerx. 2022. Models of the cardiac L-type calcium current: A quantitative review. *WIREs Mechanisms of Disease*, page e1581. (doi:10.1002/wsbm.1581)

WJ Crumb Jr, J Vicente, L Johannesen and DG Strauss. 2016. An evaluation of 30 clinical drugs against the comprehensive in vitro proarrhythmia assay (CiPA) proposed ion channel panel. *Journal of Pharmacological and Toxicological Methods*, **81**, 251–262. (doi:10.1016/j.vascn.2016.03.009)

RC Elkins, MR Davies, SJ Brough, DJ Gavaghan, Y Cui, N Abi-Gerges and GR Mirams. 2013. Variability in high-throughput ion-channel screening data and consequences for

cardiac safety assessment. *Journal of Pharmacological and Toxicological Methods*, **68**, 112–122. (doi:10.1016/j.vascn.2013.04.007)

H Guthrie, FS Livingston, U Gubler and R Garippa. 2005. A place for high-throughput electrophysiology in cardiac safety: screening hERG cell lines and novel compounds with the ion works HTTM system. *Journal of Biomolecular Screening*, **10**, 832–840. (doi:10.1177/1087057105280566)

Guideline ICH. 2006. The clinical evaluation of QT/QTc interval prolongation and proarrhythmic potential for non-antiarrhythmic drugs E14. *E14 (https://www.ich.org/page/efficacy-guidelines), accessed on 2024.04.11*, **4**.

J Kramer, CA Obejero-Paz, G Myatt, YA Kuryshev, A Bruening-Wright, JS Verducci and AM Brown. 2013. MICE models: superior to the hERG model in predicting Torsade de Pointes. *Scientific Reports*, **3**, 1–7. (doi:10.1038/srep02100)

YA Kuryshev, AM Brown, E Duzic and GE Kirsch. 2014. Evaluating state dependence and subtype selectivity of calcium channel modulators in automated electrophysiology assays. *Assay and Drug Development Technologies*, **12**, 110–119. (doi:10.1089/adt.2013.552)

G-R Li, J Feng, L Yue, M Carrier and S Nattel. 1996. Evidence for two components of delayed rectifier K^+ current in human ventricular myocytes. *Circulation Research*, **78**, 689–696. (doi:10.1161/01.RES.78.4.689)

Z Li, S Dutta, J Sheng, PN Tran, W Wu, K Chang, T Mdluli, DG Strauss and T Colatsky. 2017. Improving the in silico assessment of proarrhythmia risk by combining hERG (human ether-à-go-go-related gene) channel–drug binding kinetics and multichannel pharmacology. *Circulation: Arrhythmia and Electrophysiology*, **10**, e004628. (doi:10.1161/CIRCEP.116.004628)

Z Li, BJ Ridder, X Han, WW Wu, J Sheng, PN Tran, M Wu, A Randolph, RH Johnstone, GR Mirams *et al.* 2019. Assessment of an in silico mechanistic model for proarrhythmia risk prediction under the CiPA initiative. *Clinical Pharmacology & Therapeutics*, **105**, 466–475. (doi:10.1002/cpt.1184)

J Llopis-Lorente, J Gomis-Tena, J Cano, L Romero, J Saiz and B Trenor. 2020. In silico classifiers for the assessment of drug proarrhythmicity. *Journal of Chemical Information and Modeling*, **60**, 5172–5187. (doi:10.1021/acs.jcim.0c00201)

GR Mirams, Y Cui, A Sher, M Fink, J Cooper, BM Heath, NC McMahon, DJ Gavaghan and D Noble. 2011. Simulation of multiple ion channel block provides improved early prediction of compounds' clinical torsadogenic risk. *Cardiovascular Research*, **91**, 53–61. (doi:10.1093/cvr/cvr044)

J-I Okada, T Yoshinaga, J Kurokawa, T Washio, T Furukawa, K Sawada, S Sugiura and T Hisada. 2015. Screening system for drug-induced arrhythmogenic risk combining a patch clamp and heart simulator. *Science Advances*, **1**, e1400142. (doi:10.1126/sciadv.1400142)

WS Redfern, L Carlsson, AS Davis, WG Lynch, Il MacKenzie, S Palethorpe, PKS Siegl, I Strang, AT Sullivan, R Wallis *et al.* 2003. Relationships between preclinical cardiac electrophysiology, clinical QT interval prolongation and torsade de pointes for a broad range of drugs: evidence for a provisional safety margin in drug development. *Cardiovascular Research*, **58**, 32–45. (doi:10.1016/s0008-6363(02)00846-5)

PT Sager, G Gintant, JR Turner, S Pettit and N Stockbridge. 2014. Rechanneling the cardiac proarrhythmia safety paradigm: a meeting report from the cardiac safety research consortium. *American Heart Journal*, **167**, 292–300. (doi:10.1016/j.ahj.2013.11.004)

MC Sanguinetti, C Jiang, ME Curran and MT Keating. 1995. A mechanistic link between an inherited and an acquird cardiac arrthytmia: hERG encodes the IKr potassium channel. *Cell*, **81**, 299–307. (doi:10.1016/0092-8674(95)90340-2)

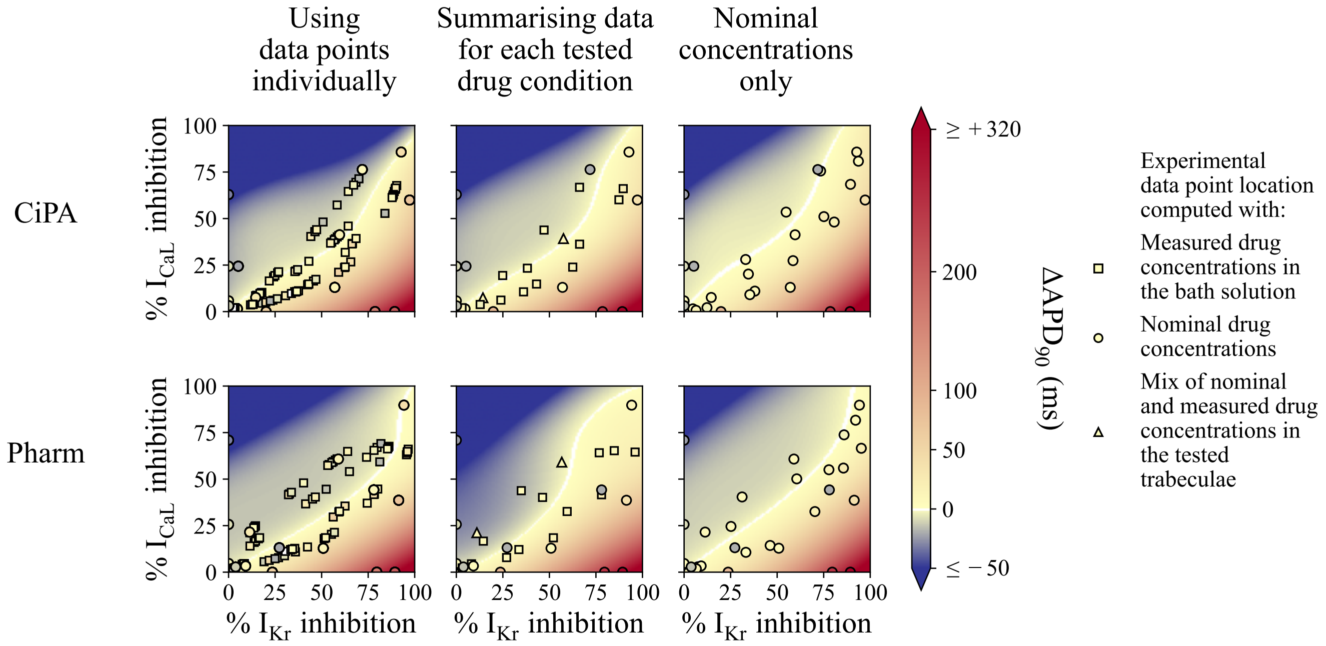

Figure D: Impact of reporting drug concentrations as measured in the bath solution or as nominal on the cubic surface and on the interpretation of the results in this study. Experimental ΔAPD_{90} measured ex-vivo under various drug conditions in human ventricular trabeculae, as a function of I_{Kr} and I_{CaL} inhibition and cubic surface approximating the experimental data points in the background. Each data point was placed with the current inhibition computed with Eq. 1 in the main text from drug concentrations and the drug IC50 for I_{Kr} and I_{CaL} (Table 2 in the main text). **Left:** ΔAPD_{90} is reported with one point per tested drug condition per trabecula. The cubic surface was fitted to all the data points for all the trabeculae. **Middle:** A single point is plotted per tested drug condition. The data for drug concentration and drug-induced ΔAPD_{90} was averaged the trabeculae tested with the same nominal concentration. **Right:** A single point is plotted per tested drug condition, using the nominal drug concentration to compute the drug-induced current inhibition and subsequent location on the map. ΔAPD_{90} was averaged similarly to the middle panel. When measured drug concentrations were available only for some trabeculae tested with the same nominal drug concentration, the data point was plotted as a **triangle**.

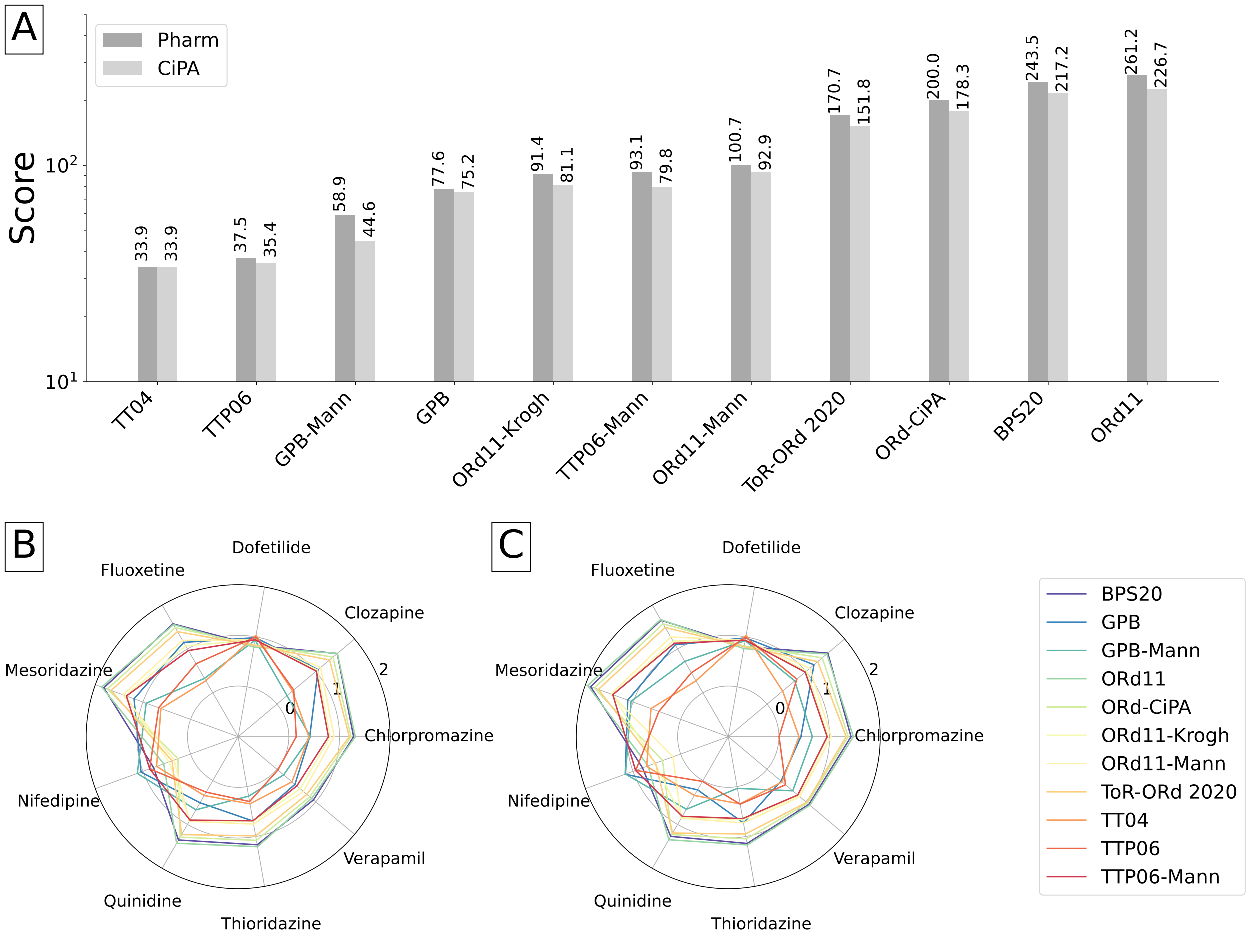

Figure E: Comparison of the abilities of human ventricular AP models to reproduce the APD$_{90}$ response to I_{Kr} and I_{CaL} inhibition observed *ex vivo*, using only nominal drug concentrations as inputs for simulations. The error metric was computed from Eq. 2 in the main text. **A:** The error measure was summed over all the drugs used in this study, when using the Pharm and CiPA protocols to compute the reduction of ionic currents by drugs. For each model, two bar plots were plotted, to compare the predictive power of models with the Pharm (left bar) and the CiPA (right bar) datasets. **B and C:** Detail of the error measures associated to each of the drugs with the CiPA and Pharm datasets, respectively, for each model. The log$_{10}$ of the error measure is plotted along the radial-axis.

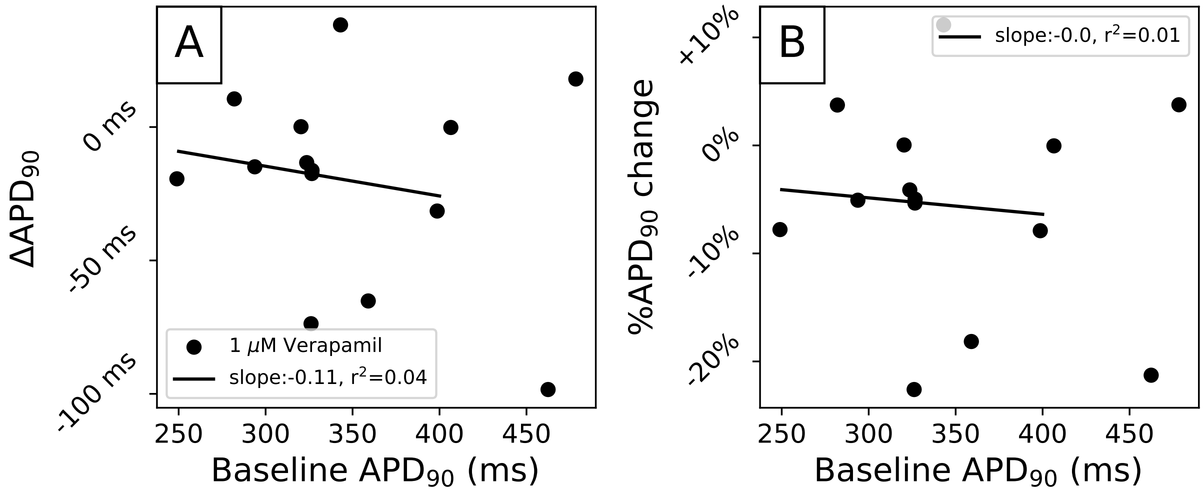

Figure F: Correlation between baseline APD_{90} and Verapamil-induced perturbations, measured as absolute change in APD_{90} (**A**) and as relative change in APD_{90} (**B**). No correlation was found.

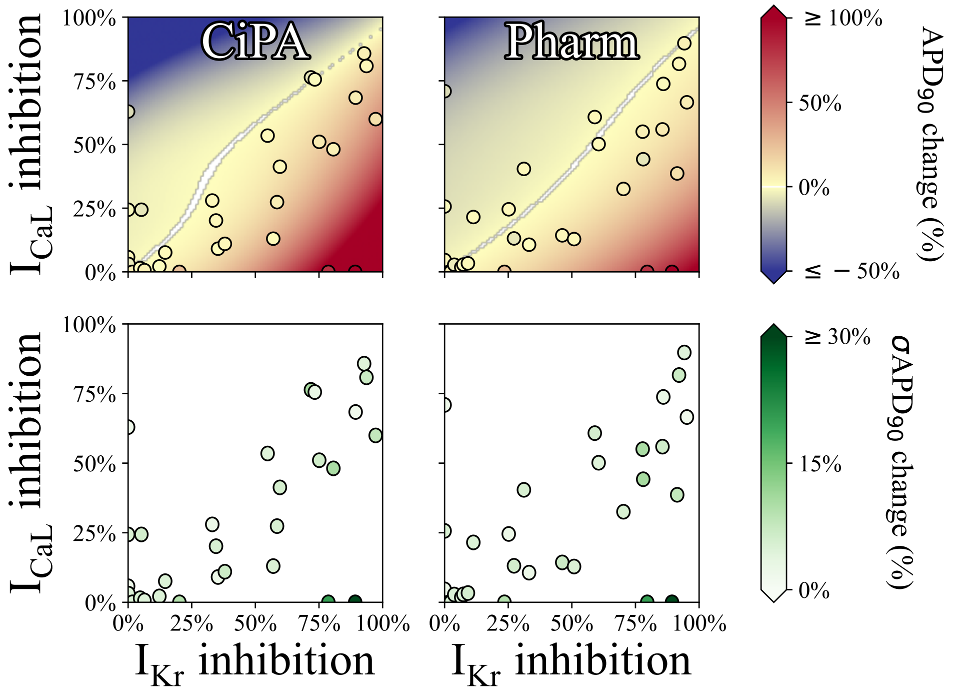

Figure G: Experimental $\%\Delta APD_{90}$ measured ex-vivo under various drug conditions in human ventricular trabeculae, as a function of I_{Kr} and I_{CaL} inhibition and cubic surface approximating the experimental data points in the background. I_{Kr} and I_{CaL} inhibition were computed using the Hill equation (Eq. 1 in the main text), with the CiPA (left) and Pharm (right) datasets (Table 2 in the main text) and nominal drug concentrations (Table 1 in the main text).

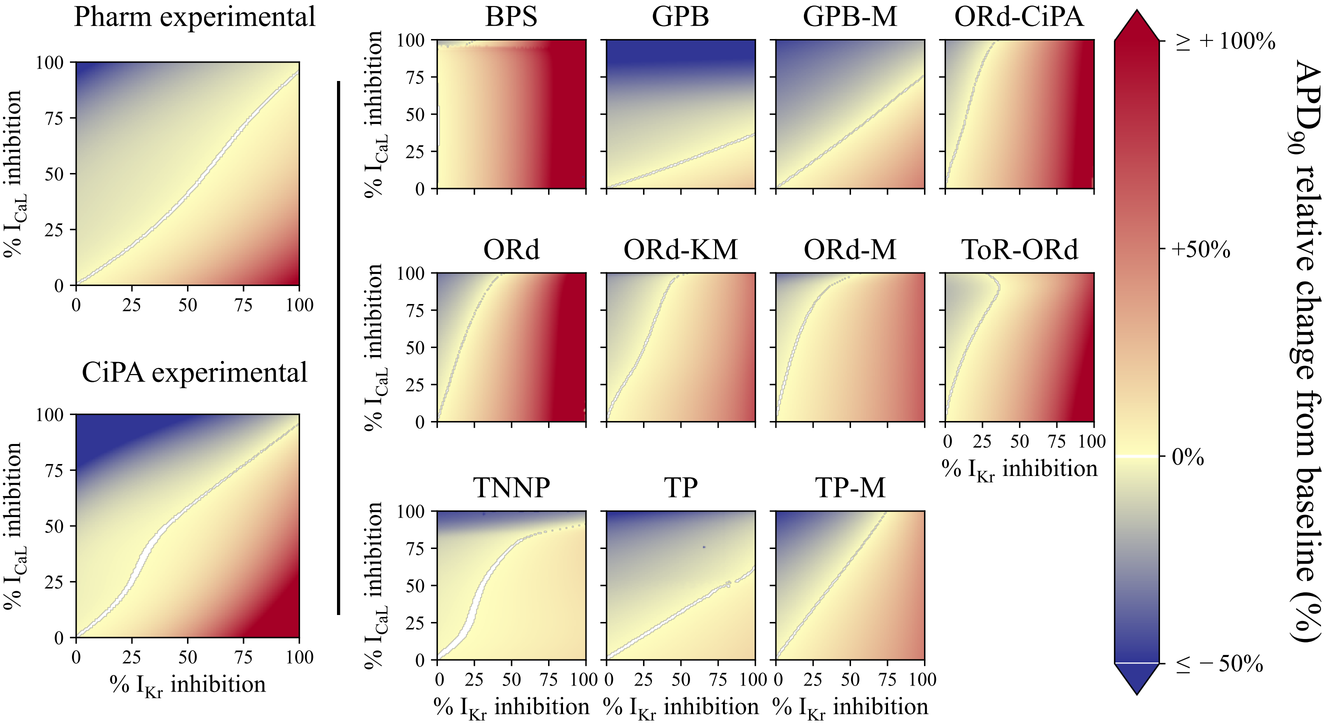

Figure H: 2-D maps of simulated $\%\Delta APD_{90}$after I_{CaL} and I_{Kr} inhibition. The colour scale indicates shortening of APD_{90} (i.e., $\Delta_{\%}APD_{90} < 0\,ms$) for colours towards dark blue, and APD_{90} prolongation (i.e., $\Delta_{\%}APD_{90} < 0\,ms$) for colours towards red. ΔAPD_{90} values below $-50\,ms$ and above $+200\,ms$ were set to dark blue and red, respectively, for better visualisation. For I_{Kr} and I_{CaL} inhibition leading to -1 ms $< \Delta APD_{90} < +1\,ms$, the pixel is coloured in white.

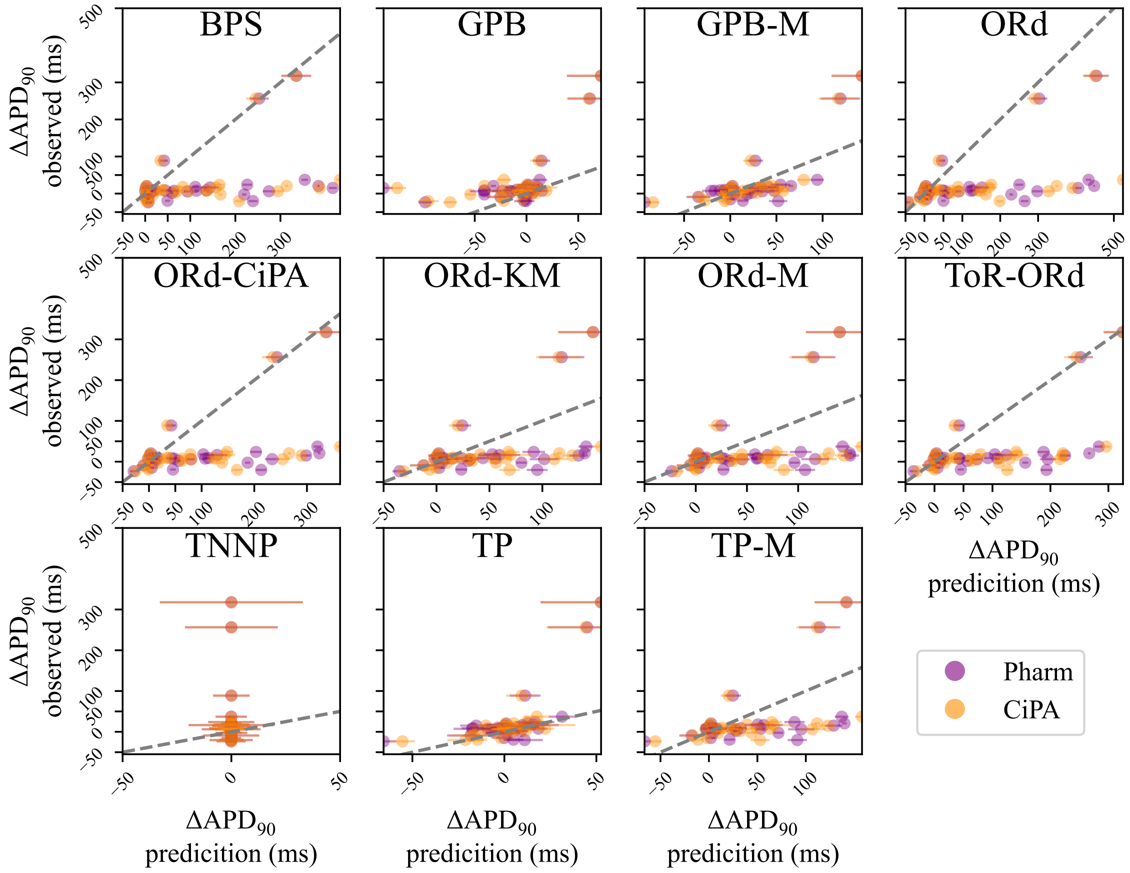

Figure I: Observed versus prediction plot for the tested models, using the CiPA and Pharm datasets (orange and purple, respectively). The experimental SEM was highlighted with horizontal error bars. The identity line is highlighted with the dashed line.

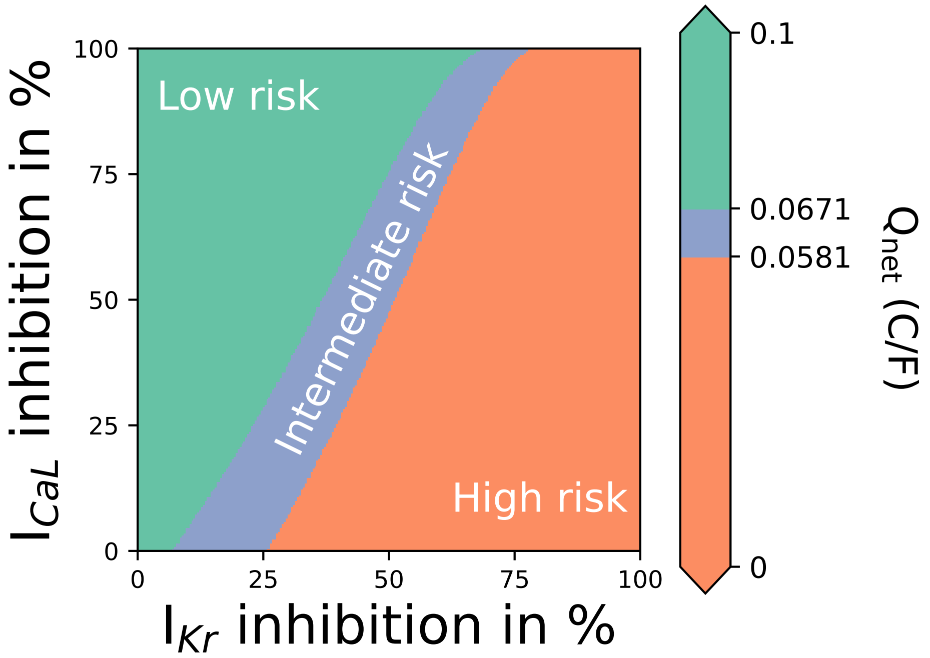

Figure J: Q_{net} computed with the ORd-CiPA model, for various combinations of I_{Kr} and/or I_{CaL} inhibition. The Torsade risk thresholds are defined in Li *et al.* (2019). Note that I_{Kr} inhibition was computed as plain reduction of the maximal conductance of I_{Kr} (Section 2.4.1 in the main text) instead of the dynamic hERG binding model used in the original study (Li *et al.*, 2019).

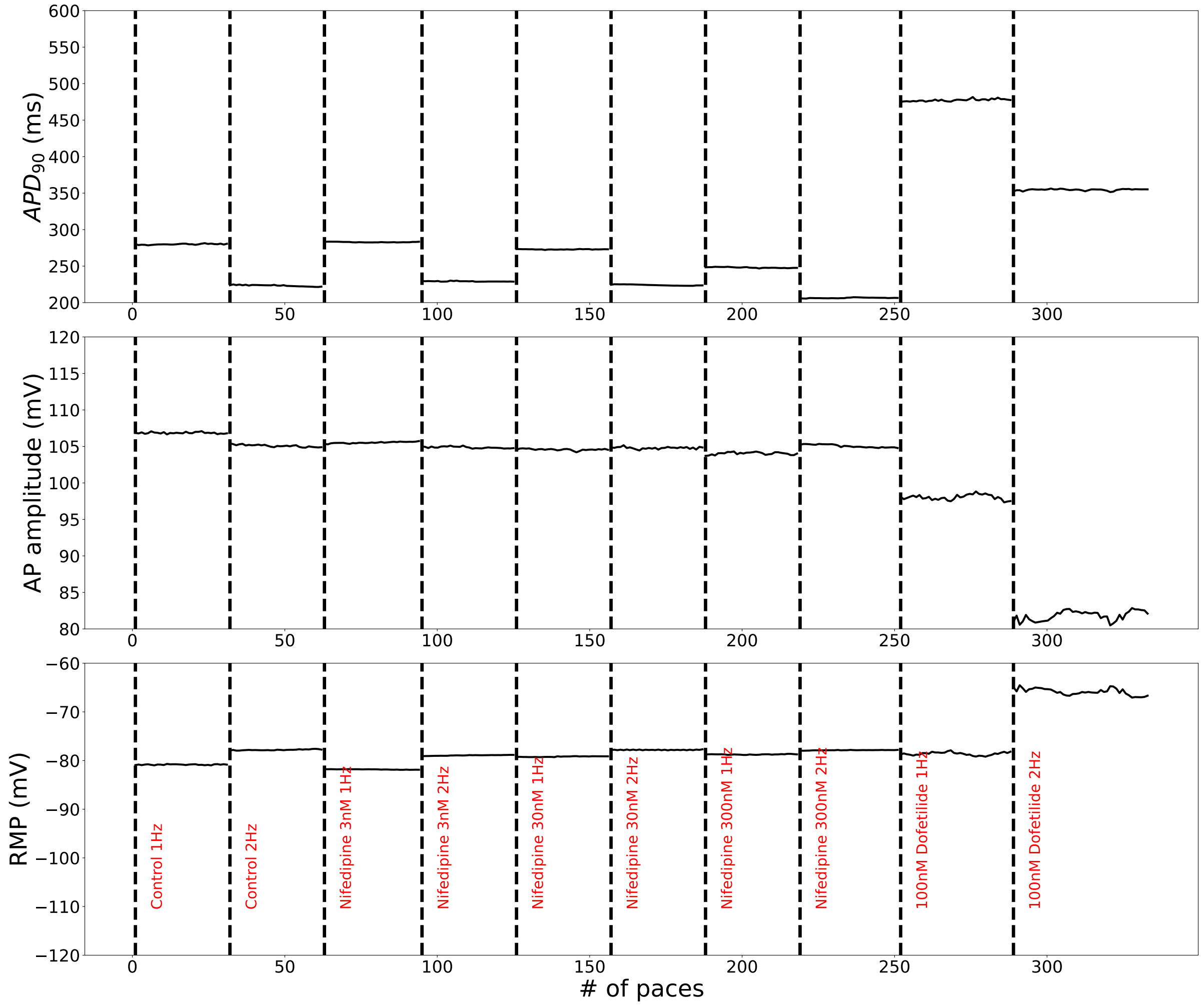

Figure K: Stability of AP markers observed in a representative trabecula, after 25 min of steady 1 Hz pacing or 3 min of 2 Hz pacing. For each condition, only the last 2 min are plotted.
